# Malware homology determination using visualized images and feature fusion

**DOI:** 10.7717/peerj-cs.494

**Published:** 2021-04-15

**Authors:** Xuejin Zhu, Jie Huang, Bin Wang, Chunyang Qi

**Affiliations:** 1 School of Cyber Science and Engineering, Southeast University, Nanjing, Jiangsu, China; 2 Purple Mountain Laboratories, Nanjing, Jiangsu, China; 3 College of Electrical Engineering, Zhejiang University, Hangzhou, Zhejiang, China

**Keywords:** Computer security, Homology determination, Malware visualization, Machine learning

## Abstract

The family homology determination of malware has become a research hotspot as the number of malware variants are on the rise. However, existing studies on malware visualization only determines homology based on the global structure features of executable, which leads creators of some malware variants with the same structure intentionally set to misclassify them as the same family. We sought to develop a homology determination method using the fusion of global structure features and local fine-grained features based on malware visualization. Specifically, the global structural information of the malware executable file was converted into a bytecode image, and the opcode semantic information of the code segment was extracted by the n-gram feature model to generate an opcode image. We also propose a dual-branch convolutional neural network, which features the opcode image and bytecode image as the final family classification basis. Our results demonstrate that the accuracy and F-measure of family homology classification based on the proposed scheme are 99.05% and 98.52% accurate, respectively, which is better than the results from a single image feature or other major schemes.

## Introduction

With the rapid development of information technology, malware has grown exponentially to become the main threat to network security. Malware generally refers to all malicious program codes, which can cause information leakage or resource abuse of the target system, devastate the integrity and availability of the system, and violate the security policy of the target system. Malware can be categorized into several types, including computer viruses, worms, Trojan horses, backdoors and logic bombs. According to the 2019 malware statistics report by McAfee Labs, the number of new malware samples intercepted in 2018 exceeded 200 million, which grew by 41.63% compared to the same period last year (McAfee, 2019). However, most of these newly discovered malware samples can be regarded as variants of existing malware and often come from the same malware family with highly similar code structures. With the increasing popularity of a variety of automated malware generation tools, attackers can easily create new variants of malicious code by modifying or confusing the code based on existing malware (Mohamed & Ithnin, 2017). Variations from the same family are called homologous malware. Analyzing the homology of malware and classifying malware accurately helps our understanding of its evolutionary trend, aids in the detection of new variants of malware, and allows us to trace their sources.

As early as 1998, Goldberg proposed the theory of constructing a virus family tree (Goldberg et al., 1998) and pioneered the study of malware homology determination. Since then, researchers have proposed many methods to determine the homology of malware, mainly based on similarities as indicators. The traditional methods can be divided into dynamic analysis and static analysis which extracts statistical features like *n*-grams or application programming interface (API) calls (Morales et al., 2010; Egele et al., 2012; Narouei et al., 2015; Catak et al., 2020). Dynamic analysis must execute malware samples in a virtual environment, and then collect and analyze behavioral information such as system calls, network operations, and registry modification records (Rieck et al., 2008; Kolosnjaji et al., 2016), to determine the homology of malware. Bailey et al. (2007) described the behavior of malware as changes of the system state at runtime, calculated the similarity by means of the standard Euclidean distance, and determined the homology by clustering. Park et al. (2010) constructed the system call graph of behavior by dynamically capturing the behavior of malware for classification and determining the code similarity. Dynamic analysis observes the actual behavior of malware, and thus can identify and classify confused and encrypted malware more effectively. It has, however, has a high system overhead and long detection cycle, causing low detection efficiency. Moreover, some malware can detect whether the running environment is a virtual environment, and then hide the real malicious intent by performing normal behavior, making it difficult to collect instances of abnormal behavior.

Considering the time and resources consumed by dynamic analysis, static analysis is more conducive to analyzing the malware and its explosively growing variants. Static homology analysis analyzes the function, code structure, and some malicious behaviors of malware without executing them. Static methods can be used for fine-grain analysis of the structure and content of code in detail, which can effectively classify the family of the malware. Qiao et al. (2013) used the system API calls sequence in malware as the basis to determine the homology of the malware and proposed a framework named CBM to dynamically restore the API after deformation to solve some code deformation problems. Santos et al. (2013) found that the statistical characteristics of the opcode sequences of malware and benign software are different, which indicated that malware can be detected by the frequency of different opcode sequences after disassembling. Zolotukhin & Hamalainen (2014) also used the *n*-gram algorithm model to extract the opcode sequence of length *n* as an input feature of malware detection. The static method does not need to actually execute the malware, so it has the advantage of high analysis efficiency. However, variants of malware usually reduce the effective code feature by means of obfuscation and deformation, resulting in low accuracy for static analysis (Moser, Kruegel & Kirda, 2007).

A new homology determination method based on malware visualization image was recently proposed. Conti et al. (2010) proposed the idea of converting binary text into a grayscale image. Nataraj et al. (2011a) then introduced this idea into the determination of malware homology, where it was proposed to convert binary executable files into grayscale images in bytes. Their results found that malware images of the same family had similar structural texture features, while the features of malware images under different families were quite different. Then, the k-nearest neighbor (KNN) algorithm was applied to compare the Gist texture features of the malware grayscale image to achieve the classification of malware samples. The introduction of malware visualization is a landmark advance in homology determination and subsequent research has made many improvements. Malware files are transformed into binary grayscale images, and then texture features are extracted by image processing technology to complete the family classification. Compared with the traditional static and dynamic methods, this method has the advantages of simple realization, low computational complexity, and high classification accuracy. It can achieve the same classification accuracy as dynamic analysis, and reduce the running time by 95% (Nataraj et al., 2011b).

Many researchers have proposed new family homology analysis methods on the basis of Nataraj method. Han et al. (2015) introduced the concept of the entropy diagram. This method does not extract texture features of the malware grayscale image, but compares the entropy value of the image. Compared to the Nataraj method, this method is able to achieve equivalent classification ability and significantly enhance efficiency. With the rapid development of deep learning in recent years, some researchers have used this method to classify malware images. Cui et al. (2018) used the convolutional neural network (CNN) to classify the grayscale images of malware, and adopted the bat algorithm to balance the number of different families. Venkatraman, Alazab & Vinayakumar (2019) proposed a hybrid architecture method based on CNN and gated recurrent unit (GRU) to classify malware images. Yuan et al. (2020) converte malware binaries into markov images according to bytes transfer probability matrixs. Then the deep CNN is used for markov images classification. Ghouti & Imam (2020) use simple algebraic dot products and support vector machines to classify malware based on representative digital images. These methods show the efficiency of deep learning in the field of malware image classification.

However, most of the existing visualization schemes (Nataraj et al., 2011a; Nataraj et al., 2011b; Han et al., 2015; Cui et al., 2018; Venkatraman, Alazab & Vinayakumar, 2019; Yuan et al., 2020; Ghouti & Imam, 2020; Chu, Liu & Zhu, 2020) convert the whole portable executable (PE) format binary malware file into an image and the feature information contained in the image is typically contained in the global structure of the file. The code segment image only takes a small part of the whole malware image, and is a compiled “black box feature”, which leads to the neglect of the specific semantic information contained in the code segment. This flaw will cause malwares with a similar file global structure to be mistakenly classified into the same family, which, in fact, do not belong to the same family. Malware makers may also deliberately change their own file structural features to avoid detection. Therefore, it is not enough to analyze the homology of malware based on the binary image. From existing static analysis research, we can see that the semantic information of the code segment, called the “white box feature”, should be considered as the most important basis to represent a malware feature (Gandotra, Bansal & Sofat, 2014). Compared with other existing works of malware homology determination based on visualization, our method makes the malware feature information more specific. The targeted semantic information features of the code segment are extracted and converted into the opcode image while using the whole binary byte grayscale image.

A novel visualization method based on feature fusion is proposed for the malware homology determination in this paper. The main contributions of this work are summarized as follows:

(1) We transformed global structural features of the malware PE file into a bytecode image and designed a construction method of the opcode image. The specific semantic features contained in the code segment of the PE file were extracted and transformed into the opcode image so as to make the malware features more specific.

(2) We proposed a dual-branch CNN model to fuse the features of the bytecode image and opcode image to get an accurate and efficient malware family classification.

(3) The experimental results show that this method combines the global structure and local semantic features of malware, and had a higher classification performance than single feature or other major methods.

## Materials & methods

**Figure 1:**
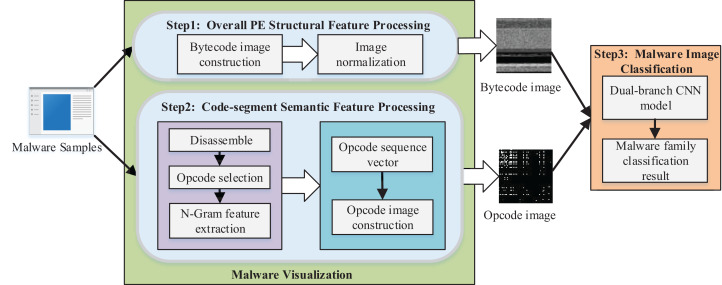
Overview of the proposed method.

We proposed a method of malware homology determination based on malware visualization and malware image classification. The overall architecture of the scheme is shown in Fig. 1.

We converted the PE file of malware to a bytecode grayscale image with 8-bit as a unit for malware visualization. We propose the opcode image, which is used to specify the code segment information since the bytecode image mainly contains the global structure features of malware and lacks the semantic information features of code segments. First, we disassembled the PE file to access the assembly file of malware and selected the most critical m opcodes as the feature analysis. Then the *n*-gram model was applied to extract the sequence of opcodes as the feature, and each malware sample was transformed to an $m^{2}$-dimensional opcode sequence vector. The weight in the direction of each opcode sequence in the vector was represented by the product of frequency and information gain rate. Finally, we transformed the $m^{2}$-dimensional opcode sequence vector into the m×m opcode grayscale image.

We designed a dual-branch CNN classification model for feature fusion, which took the bytecode image and opcode image as the input of two branch networks to extract the deep features of the malware image for malware image classification. Then, the feature maps of the branch networks were fused and used as the final classification basis of the Softmax classifier in the network to obtain the malware classification model with specific features.

### Malware visualization

We propose an improved method of malware visualization, including (1) converting the PE file to a bytecode grayscale image; and (2) extracting the semantic information from the PE file code segment and converting it to an opcode grayscale image.

#### Bytecode image construction

**Figure 2:**
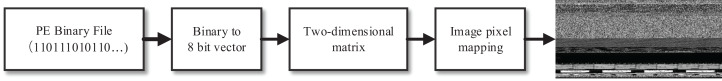
Construction process of bytecode image.

The process of converting a given executable file of malware, i.e., a PE binary file, to an image is shown in Fig. 2. First, the 8-bit length was used as the unit to read in turn and converted it into an integer in the range of 0 to 255, thus forming a one-dimensional vector. Then, according to the fixed row width, we transformed it into a two-dimensional matrix. The value range of each element in the matrix was the same as the image pixel value, which was 0 to 255. This allowed the elements in the matrix to be mapped as pixels, further converted into grayscale image, which we called bytecode images.

We set the width of the bytecode image to a fixed value, while the height depended on the PE file size. Since the sizes of different malware files varied greatly, we adopted different image widths for different file sizes, as shown in Table 1.

**Table 1:** Bytecode grayscale image size.

PE file size	Image width
<1,024 kb	512
1,024–4,096 kb	1,024
>4,096 kb	2,048

#### Opcode image construction

We obtained the global structure information of the malware PE file after retrieving the bytecode image. We then specified the code segment of PE file and converted the opcode feature information into an opcode image after disassembling the code segment.

The PE file of malware uses flat address space, so the code and data are stored in different blocks in a more fixed format. For example, the code segment is specially applied to store the program code of PE file. Although the information contained in the segment has certain redundancy, it is obviously the most representative feature in the malware information. Therefore, we used the assembly code features of this part to analyze the homology of the malware.

**Figure 3:**
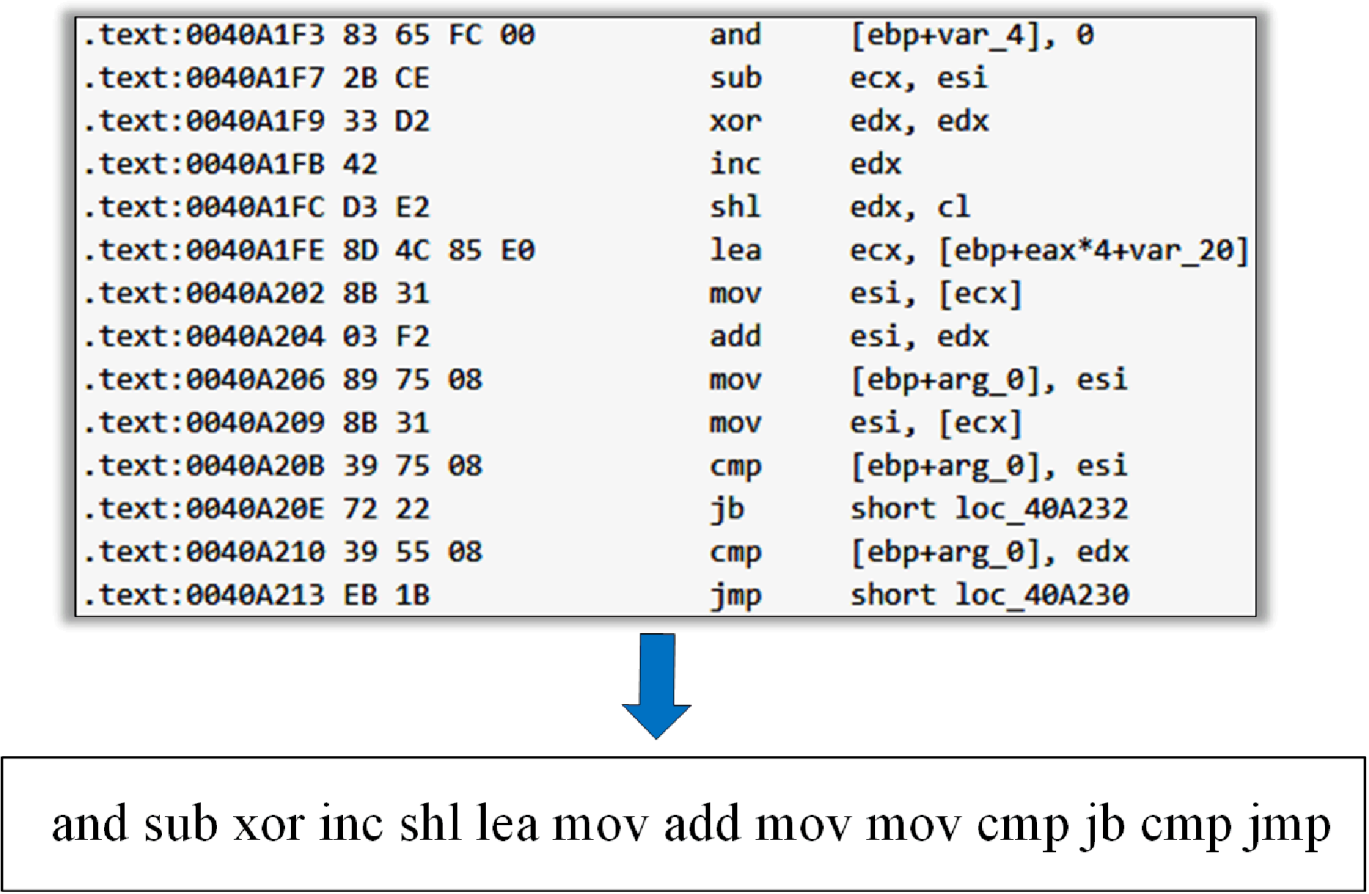
Extraction of the opcode stream from malware assembly file.

We disassembled the malware sample in the PE format through IDA Pro, which generated the “.asm” format assembly file. In assembly file text, “.text” or “CODE” was typically used to identify each line of code instruction, where the instruction structure contained the opcodes and operands. The operand is actually the memory address of a function or variable. The malware variants with homology were obtained by a deforming code, which may cause operands to change. The part of the opcode in the instruction was unchanged or rarely changed in malware deformation (Bilar, 2007). Therefore, we only used the opcode as the feature and did not consider the operands. We extracted the opcode from the assembly code in the malware assembly file to get the opcode stream (Fig. 3).

It should be noted that malware developers may use techniques such as code obfuscation to disassemble, so that the disassembled program contains invalid interference instructions or control flow graphs. However, the method proposed in this paper mainly focuses on the statistical characteristics of each opcode sequence in malicious code, and it is difficult for code obfuscation techniques to completely change this characteristic.

N-gram is a commonly used semantic feature model in natural language processing, which considers that the occurrence probability of the *n*^th^ word is only related to the preceding *n−*1 word. Program code is essentially a text language, such as the assembly code obtained above also has language structural and semantic features, so the n-gram model was used as the feature analysis and extraction method of malware. We introduced the n-gram model into the semantic information analysis of opcodes, and previous studies have shown that different malware families have different *n*-gram characteristics (Santos et al., 2009; Li, Chen & Cui, 2017).

However, before extracting the n-gram features, we considered that there are many very rare opcodes in the opcode stream of malware, and the n-gram sequence formed by them only appears in a few malware and may be used by malware makers for code obfuscation. Therefore, these opcodes did not contribute to our analysis because of their capacity for rapid growth of the feature. In order to avoid the influence of rare opcodes on the n-gram feature capacity, we first selected the key m opcodes according to their probability before extracting the opcode sequence. For the set O composed of all the opcodes in the training set *X*, the probability of the set element $o_{i}$ is calculated as follows:

(1) $$p\left(o_{i}\right)=\frac{\sum_{x_{i}\in X}freq\left(o_{i}|x_{i}\right)}{\sum_{o_{i}\in O}\sum_{x_{i}\in X}freq\left(o_{i}|x_{i}\right)}$$

where $freq\left(o_{i}|x_{i}\right)$ refers to the frequency of opcode $o_{i}$ in the sample $x_{i}$ that belongs to the training set X. For the opcode stream extracted from specific malware samples, we deleted the non-critical rare opcode to get a simplified opcode stream. In this way, we greatly reduced the *n*-gram feature capacity, so as to improve the calculation efficiency for the subsequent analysis.

For the simplified opcode stream, we use a sliding window with the size of 2 to extract a series of 2-gram opcode sequences, and then counted the frequency of each sequence. In this paper, we set the sequence length of opcodes to 2 instead of using a single opcode, because most operations with malicious intent in malware need to be completed by consecutive multiple opcodes. However, if we extracted a larger length of the opcode sequence, the number of features would be greatly increased, resulting in a rapid increase in computational overhead.

For some opcode stream of malware samples (Fig. 2), if the opcode ‘*shl*’ does not belong to the m key opcodes screened, the final n-gram opcode sequence generated is $os_{1}=\left(and,sub\right),$ $os_{2}=\left(sub,xor\right),os_{3}=\left(xor,inc\right)$…$\;os_{12}=\left(cmp,jmp\right)$, and the frequency of each sequence is 1.

We can construct $m^{2}$ opcode sequences with the length 2 by means of the selected m key opcodes according to permutations and combinations in pairs, and then assign a weight factor $w_{i}$ to each sequence $os_{i}$ to get a $m^{2}$-dimension opcode sequence vector:

(2) $$V=\left\{\left(os_{1},w_{1}\right),\left(os_{2},w_{2}\right),\ldots ,\left(os_{m^{2}},w_{m^{2}}\right)\right\}$$

The weight factor $w_{i}\;$is calculated by a new computing method called *TF-GR*, and is equal to the product of the word relative frequency $\;tf_{i}$ of the opcode sequence and the information gain rate:

(3) $$w_{i}=tf_{i}\times GR\left(X,\;os_{i}\right)$$

where $GR\left(X,os_{i}\right)$ represents the information gain rate of the opcode sequence $os_{i}\;$ to the malware training set X. If there are l opcode sequences in a sample of malware, the frequency of occurrence of each $os_{i}$ is denoted by $n_{i}$. The$\;tf_{i}\;$and $GR\left(X,os_{i}\right)$ of each $os_{i}$ for a malware simple are calculated as follows:

(4) $$tf_{i}=\frac{n_{i}}{\sum_{j=1}^{l}n_{j}}$$

(5) $$GR\left(X,os_{i}\right)=\frac{H\left(X\right)-H\left(X|os_{i}\right)}{H\left(os_{i}\right)}$$

where H(X)and $H(os_{i})\;$represent the information entropy of dataset *X* and opcode sequence $os_{i}\;$respectively, $H(X|os_{i})\;$represents the conditional entropy of dataset *X* under opcode sequence $os_{i}$. If the data set *X* has *k* different families $f_{c}$, c=1,2,…,k, and the number of opcode sequence $os_{i}$ appears in all malware samples has *s* different values $num_{j}\left(os_{i}\right)$, j=1,2,…,s, then H(X), $H(os_{i})$ and $H(X|os_{i})$ can be expressed as follows:

(6) $$H\left(X\right)=-\overset{k}{\underset{c=1}{\sum }}p\left(f_{c}\right)\log_{2}p\left(f_{c}\right)$$

(7) $$H\left(X|os{}_{i}\right)=\overset{s}{\underset{j=1}{\sum }}p\left(num{}_{j}\left(os{}_{i}\right)\right)H\left(X\;|\;num\left(os_{i}\right)=num{}_{j}\left(os{}_{i}\right)\right)$$

(8) $$H\left(os{}_{i}\right)=-\overset{s}{\underset{j=1}{\sum }}p\left(num{}_{j}\left(os{}_{i}\right)\right)\log_{2}p\left(num{}_{j}\left(os{}_{i}\right)\right)$$

It is important to note that if a malware sample does not contain a specific opcode sequence, the weight factor of the opcode sequence is equal to 0, because its frequency is 0.

In this subsection we converted the opcode sequence vector ***V*** obtained previously into a grayscale image. We took m selected key opcodes as the horizontal axis and the vertical axis of the image respectively, for images regarded as a pixel matrix and then constructed an m×m opcode image matrix. The two-dimensional coordinates of each point in the matrix were expressed as an opcode sequence with the length 2, as shown in Table 2. Then, we mapped the opcode sequence vector to the image. The sequence in each element of the vector was mapped to the position of a specific pixel point in the image matrix, and the weight factor corresponding to the sequence was expressed as the pixel value of the point.

**Table 2:** Opcode image matrix.

	$opcode_{1}$	$opcode_{2}$	$opcode_{3}$	…	$opcode_{1}$
$opcode_{1}$	$\left(o_{1},o_{1}\right)$	$\left(o_{1},o_{2}\right)$	$\left(o_{1},o_{3}\right)$	…	$\left(o_{1},o_{m}\right)$
$opcode_{2}$	$\left(o_{2},o_{1}\right)$	$\left(o_{2},o_{2}\right)$	$\left(o_{2},o_{3}\right)$	…	$\left(o_{2},o_{m}\right)$
$opcode_{3}$	$\left(o_{3},o_{1}\right)$	$\left(o_{3},o_{2}\right)$	$\left(o_{3},o_{3}\right)$	…	$\left(o_{3},o_{m}\right)$
…	…	…	…	…	…
$opcode_{m}$	$\left(o_{m},o_{1}\right)$	$\left(o_{m},o_{2}\right)$	$\left(o_{m},o_{3}\right)$	…	$\left(o_{m},o_{m}\right)$

Because the pixel value of the image is between the interval [0 255], we normalized the weight factor so that its value fell within the range of pixel value as follows:

(9) $$pix_{i}=\frac{w_{i}}{max\left(w_{i}\right)}\times 255$$

**Figure 4:**
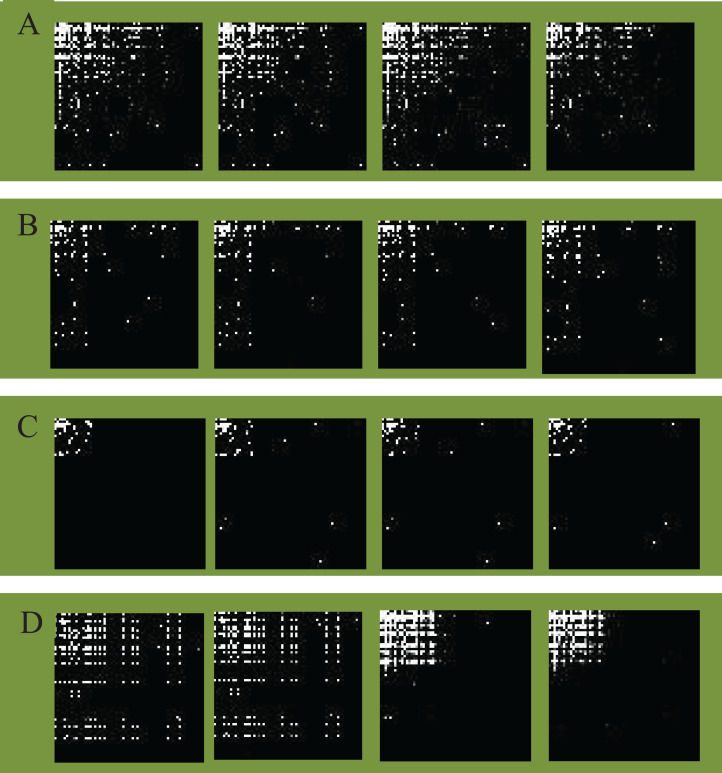
Opcode images of different families. (A) Lollipop family. (B) Kelihos_ver3 family. (C) Kelihos_ver1 family. (D) Gatak family.

According to the above grayscale image generation method, we mapped the malware opcode sequence vectors of different families to opcode images. The detailed opcode image construction process is given in Algorithm 1. Figure 4 illustrates an example of different families of opcode images (image size 64×64). Malware opcode images of different families are visually distinct, while malware with homology has a high visual similarity in opcode images due to the similar statistical characteristics of opcode.

**Algorithm 1:** Opcode image construction.

**Input:** *files:* malware assembly files; **Output:** *images:* pixel value of all opcode images;
1: **for** each file *f* in files
2: *seq* ← **get_opcode_seq** (*f*);
3: ***V*** ← **get_seq_vector** (*seq*);
4: Initialize an m×m matrix: *image*;
5: **for** each element (os,w) in ***V* do**
6: (i,j) ← **map** (os);
7: **if** w!=0 **then**
8: *image*(i,j)=**normalization**$\;\left(w_{i}\right)$;
9: **else**
10: *image*(i,j)=0;
11: **end for**
12: *images*.**add**(*image*);
13: **end for**
14: **return**(*images*);

### Classification of malware image based on CNN

As a classical deep neural network, CNN is widely used in the field of computer vision and speech processing. Compared with the general fully-connected neural network, CNN mainly uses weight sharing, local sensing, and down-sampling techniques to greatly reduce the number of weight parameters, thus significantly reducing the computation in model training. Meanwhile, CNN directly takes the original image as the input, and the convolution layer is responsible for the extraction of image features. As a result, there is no need for additional feature extraction. Each convolution layer outputs a set of feature graphs, and each feature graph represents a higher-order feature extracted by a specific filter. We propose a dual-branch CNN model suitable for the classification of malware images, which fuse the features of the bytecode image and opcode image to produce classification results.

#### Image preprocessing

We preprocessed the malware image to meet the requirements of CNN for input data. The CNN model is required to input image data of the same size when it performs a task such as image classification. Moreover, in order to facilitate the subsequent convolution operation, the length and width of image data should be the same. Due to the different sizes of executable PE files of malware, the sizes of various converted bytecode images are also different. Therefore, it is necessary to normalize all grayscale images.

**Figure 5:**
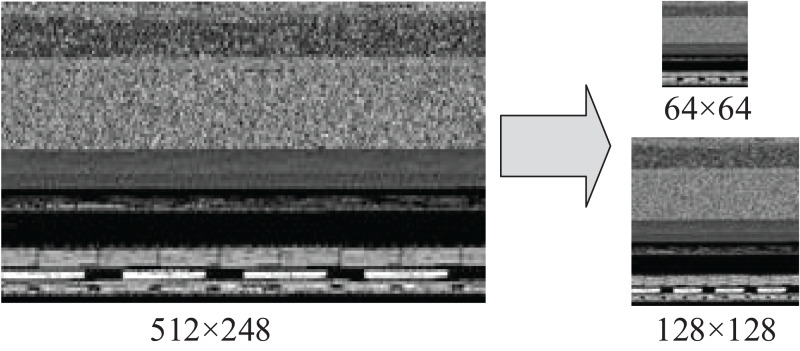
Reshape the bytecode image to a fixed size square image.

This paper uses four pixel values of the nearest neighbor in the original image to determine a pixel value of the target image by means of bilinear interpolation algorithm, which does not produce the sawtooth effect. The algorithm is more suitable for our scenario than the nearest neighbor interpolation. The normalized size of the grayscale image is a hyper-parameter, reflecting the relationship between classification accuracy and computational expense (Zhang & Wu, 2006). The larger the normalized image size, the more information CNN input data contains, resulting in better classification effect at a cost of higher time consumption. However, the bytecode image and opcode image need to be input to CNN as two channels at the same time. In order to facilitate the subsequent feature fusion operation, we kept the two input data formats unified. The size of the opcode image was m×m, so the bytecode image also needed to get the grayscale image of m×m through sampling operation. Figure 5 shows a sample of a byte grayscale image with the original size of 512 × 248 converted into 128 × 128 and 64 × 64 input images after sampling.

#### Dual-branch CNN model design

**Figure 6:**
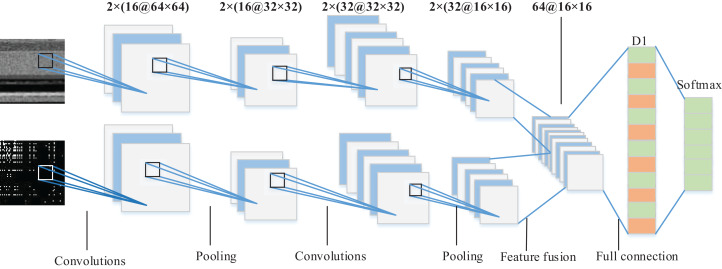
Architecture of the proposed dual-branch CNN model.

We propose a dual-branch CNN model suitable for malware image classification as shown in Fig. 6. Bytecode image and opcode image are used respectively as input of dual-tunnel branch networks of the CNN model to extract the deep features of malware. Then, the feature maps respectively obtained by the two branch CNN networks were fused in the last fully-connected layer which served as the final classification basis of the Softmax classifier. Specifically, each branch network consisted of two convolution layers with the ReLU activation function. Their filter size was 3 × 3 and the number is 16 and 32, respectively. Each convolution layer was followed by a max pooling layer, where the pooling size was 2 × 2 and stride was 2. Figure 6 shows a 64 × 64 figure as an example, and 32 feature maps with the size of 16 × 16 were output from each branch network after two rounds of convolution feature extraction. We fused the feature maps to get 64 feature maps. Since the output of the pooling layer was multidimensional, it was necessary to use the flattened layer to convert multidimensional nodes into one-dimensional nodes as the input of the fully-connected layer D1 with 512 nodes. In order to prevent over-fitting, we also added the dropout layer after the fully-connected D1 layer. The final part of the whole network model was the Softmax layer to output the family classification results. The specificity of dual-branch CNN structure and parameters is shown in Table 3.

**Table 3:** The list of dual-branch CNN structure parameters.

Network layer type	Size	Output dimension
Input layer	–	(2, 1, 64, 64)
Convolutional layer	2 × (16 @ 3 × 3) filter	(2, 16, 64, 64)
Max pooling layer	2 × 2, stride 2	(2, 16, 32, 32)
Convolutional layer	2 × (32 @ 3 × 3) filter	(2, 32, 32, 32)
Max pooling layer	2 × 2, stride 2	(2, 32, 16, 16)
Fully connected layer	512 nodes	(512,1)
Output layer	–	1

Convolution maps each position of the image to a new value through a linear transformation to extract a simple feature of the data. Multi-layer convolution uses layer-by-layer mapping to extract more complex abstract features of the image; the feature map may be obtained using the activation function after convolution. The process of convolution operation can be expressed as follows:

(10) $$h\left(i\right)=f\left(\sum_{x=1}^{n}\sum_{y=1}^{n}\sum_{z=1}^{m}a_{x,y,z}\times w_{x,y,j}^{i}+b^{i}\right)$$

where $a_{x,y,z}$ represents the value of input data node (x,y,z) in the filter, and $w_{x,y,j}^{i}$ represents the weight of the filter for the input node (x,y,z). $b^{i}$ represents the bias parameter corresponding to the output node. f() is the activation function. n represents the length and width of the filter, and m indicates the depth of the filter.

The pooling layer was used for image feature downsampling and feature dimension reduction. The data processing capacity was compressed while the effective information was retained, which effectively reduced over-fitting and improved the fault tolerance of the model. For the input feature map, new smaller size features were obtained through the pooling operation of Formula (11).

(11) $$Y_{j}^{i+1}=downsamp\left(Y_{j}^{i}\right)+b_{j}^{i+1}$$

where downsamp(·) represents the downsampling mode under image features, and $b_{j}^{i+1}$ represents the bias parameter. In general, max-pooling or mean-pooling can be selected for pixels in the field determined by the filter. We found that the pooling layer of the CNN adopts the max pooling image feature subsampling method.

## Experiments and results

### Experimental data

In order to verify the effectiveness of our proposed method, we used real malware dataset (Big 2015 provided by Microsoft) as the experimental data (Kaggle, 2015). Since the dataset does not publish the family label of the malware in the test set, we only use the malware samples in the training set. The training set of the dataset contains 10,868 malware program samples from nine families. Each malware sample in the dataset has a binary representation of the executable file and its corresponding disassembly file. It should be noted that, since the disassembly file is directly provided in the malware samples, there was no need to use IDA Pro to perform the disassembly operation during the experiments. Table 4 shows the family distribution of malware programs present in the dataset.

**Table 4:** The sample distribution of the dataset.

Family	Sample number	Family ID
Ramnit	1,541	1
Lollipop	2,478	2
Kelihos_ver3	2,942	3
Vundo	475	4
Simda	42	5
Tracur	751	6
Kelihos_ver1	398	7
Obfuscator.ACY	1,228	8
Gatak	1,013	9

### Experimental design

The experimental environment adopted Intel i7-9700 CPU @ 3.00 GHz, with 32.0 GB of RAM, and the GPU was GeForce RTX 2060S. Tensorflow was used to conduct deep learning classification of malware images for the dataset.

We tested different hyper-parameters to determine the best classification model. The hyper-parameters to tune the dual-branch CNN model are listed in Table 5. We adjusted the hyper-parameters in the search space, and then found the best value. For example, when the number of epoch exceeded 20, the model test results did not continue to improve, and the training time was longer, so this hyper-parameter was set to 20. The learning rate was another important hyper-parameter, which determined whether the objective function converges to a local minimum and when. A proper learning rate can make the objective function converge to a local minimum in proper time. We set the initial learning rate to 0.0013. In addition, we set the decay of learning rate to 0.02. The learning rate gradually decreased as the number of iterations increased, which sped up training in the early stages of training. The learning rate during each iteration of training is as follows:

**Table 5:** Hyper-parameters to tune the dual-branch CNN model.

Hyper-parameter	Search space	Best value
Epoch	10–50	20
Batch training samples	64–512	128
Optimization algorithm	SGD, Adadelta, RMSprop, Adam, Adagrad	Adam
Initial learning rate	0.0001–0.01	0.0013
Decay of learning rate	0.001–0.1	0.02
Dropout probability	0.1–0.9	0.5
Loss function	–	categorical_crossentropy

(12) $$lr_{iteration\;}=lr_{iteration\;-1}\times \frac{1}{1+decay\times iteration\;}$$

where $lr_{iteration\;}$ represents the learning rate at the iteration^th^. In addition, the classification of malware families is a multi-classification problem, so we used categorical cross-entropy as the loss function.

The accuracy measure was used to evaluate the overall classification performance, which is essentially the number of correctly classified samples divided by the total number of samples. In addition, in order to comprehensively evaluate our model, additional evaluation metrics were adopted, including precision, recall and F-measure. Accuracy is often misleading and using accuracy metrics alone can lead to one-sided results. This situation occurs when the class imbalance is large, that is, the predicted results of the model for all tasks are of the majority class, and the overall classification accuracy is high, but the predictive ability of the minority class is poor (Gibert et al., 2019). Therefore, it may be better to select a model that has lower accuracy but greater predictive ability of the problem in some scenarios. The evaluation metrics are as follows:

(13) $$Accuracy=\frac{\sum_{i\in C}TP{}_{i}}{N}$$

(14) $$Precision=\frac{\sum_{i\in C}TP{}_{i}}{\sum_{i\in C}(TP{}_{i}+FP{}_{i})}$$

(15) $$Recall=\frac{\sum_{i\in C}TP{}_{i}}{\sum_{i\in C}\left(TP{}_{i}+FN{}_{i}\right)}$$

(16) $$F-measure=\frac{2\times Reacll\times Precision}{Reacll+Precision}$$

where N refers to the total number of samples in the dataset with k families. The parameters such as ${TP}_{i}$ (True Positive), ${TN}_{i}$ (True Negative), ${FP}_{i}$ (False Positive) and ${FN}_{i}$ (False Negative) are usually defined in binary classification problems. In this paper, since our target task is a multi-class classification task with the malware families set C, the definition of these parameters for a specific malware family i∈C are shown as follows:

- ${TP}_{i}$ refers to the number of samples classified as familyi and actually belong to family i.
- ${TN}_{i}$ refers to the number of samples not classified as family i and actually do not belong to family i.
- ${FP}_{i}$ refers to the number of samples classified as family i but actually do not belong to family i.
- ${FN}_{i}$ refers to the number of samples not classified as family i but actually belong to the family i.

In order to better evaluate our model, we used 10-fold cross validation. The dataset was divided into 10 sub-samples. In each experiment, one sub-sample was used as the validation set and the other nine sub-samples were used as the training set. The training and testing of the model were performed in a loop 10 times, and then the average of the results was taken as the final evaluation.

### Effects of image size on classification performance

**Figure 7:**
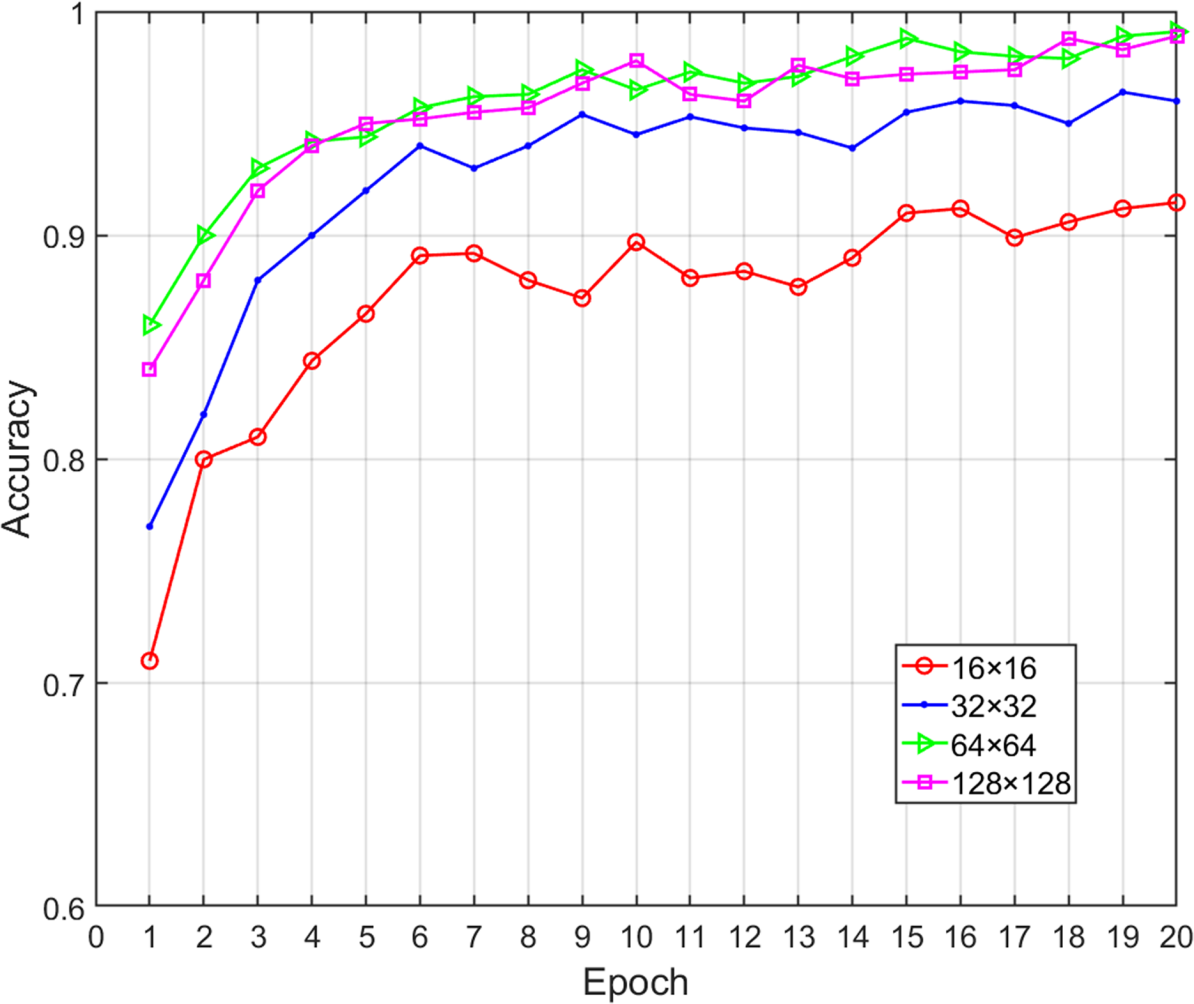
Accuracy of malware classification with different size of image.

In our proposed method, the opcode image was composed of m key opcodes, which means that the value of m was critical to the performance of the proposed model. A lower value probably did not retain enough the important feature information (i.e., lost discriminative information for a family) while higher values only increased the computational time without increasing the classification accuracy. When m is equal to 16, 32, 64 and 128, respectively, the corresponding opcode image size will be 16 × 16, 32 × 32, 64 × 64, 128 × 128. At the same time, the bytecode image was sampled to make it consistent with the size of opcode image. We obtained various performance metrics of sample classification under different m sizes through experiments (Table 6). Figure 7 shows the change of classification accuracy with the increase of training epochs under different image sizes. It can be seen that after 20 epochs of model training, the classification performance achieved by 64 × 64 and 128 × 128 was highly similar. When m was greater than 64, the performance did not have a positive correlation with the value of m, and the time overhead was rapid growth. Therefore, in order to reduce the number of calculations, the value of m should be fixed as 64. Malware images from the Big 2015 dataset were sampled to corresponding 64 × 64 pixels and the greater width and height did not improve the performance of the classification model.

**Table 6:** Effects of image size on classification performance.

Image size	Accuracy	Precision	Recall	F1-measure	Time (ms)
16 × 16	0.9154	0.9037	0.8934	0.8985	12
32 × 32	0.9641	0.9630	0.9672	0.9651	30
64 × 64	0.9905	0.9871	0.9833	0.9852	42
128 × 128	0.9907	0.9846	0.9812	0.9829	180

### Effects of feature description on classification performance

**Figure 8:**
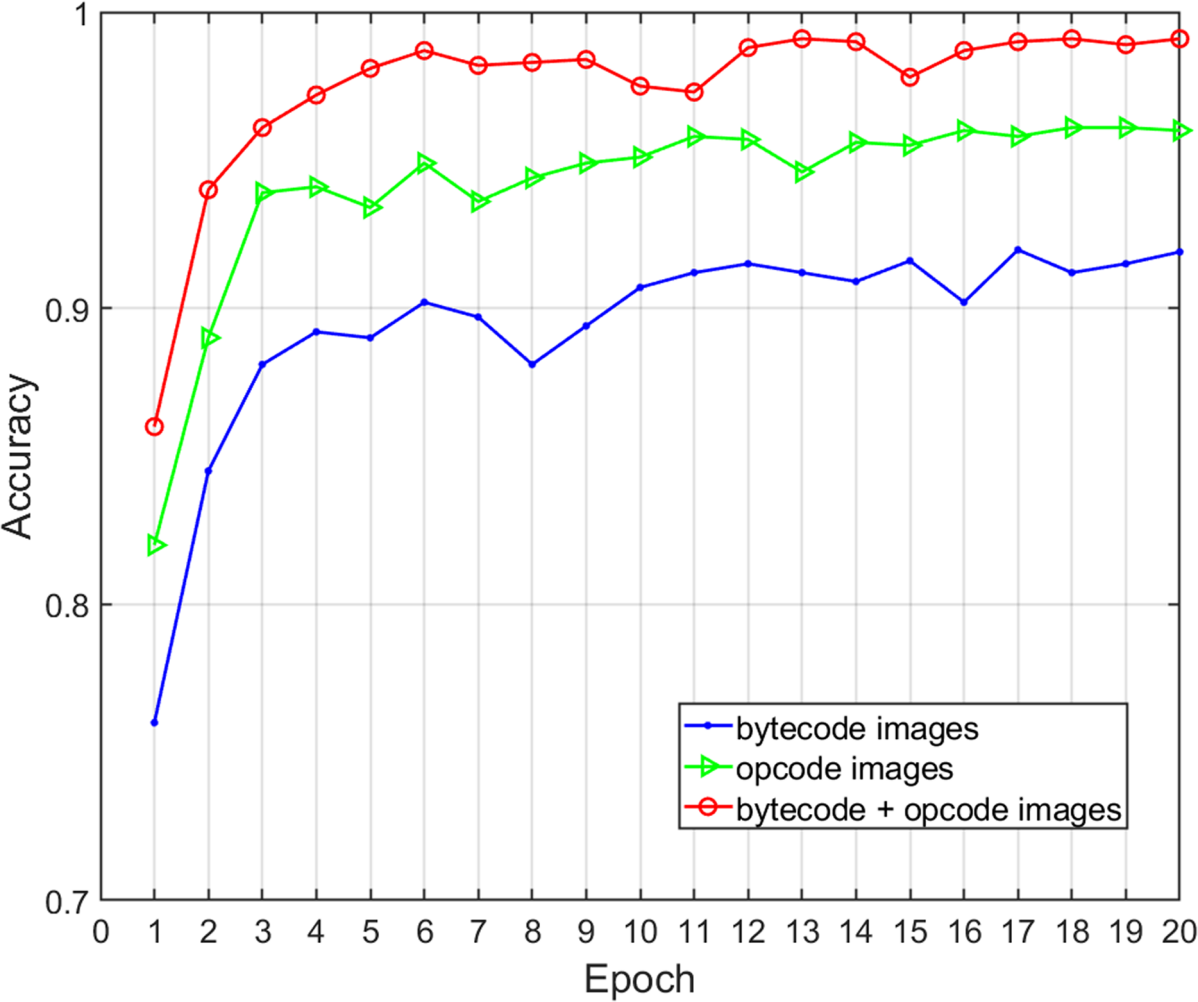
Accuracy of malware classification with different feature descriptions.

Three different feature selections were adopted to assess the effects of the malware feature description on the family classification performance and time overhead of the proposed method, including global structural features (bytecode image), local semantic features (opcode image), and structural and semantic features combined (bytecode and opcode images). We obtained various performance metrics of sample classification under three different feature descriptions through experiments (Table 7). Figure 8 shows the change of classification accuracy with the increase of training epochs under different feature combinations. The results show that the combination of structural and semantic features was superior to individual structural features or individual semantic features in classification performance. In terms of the single feature, the accuracy and F-measure of the semantic features were 4.16% and 3.44% higher than structural features respectively, so the proposed opcode image was more effective than the bytecode image for malware classification. In terms of the fusion of semantic features and structural features, the accuracy and F-measure improved by 7.04% and 6.34% when compared with that of structural features only, and the length of time was acceptable. Therefore, this experiment proves that the addition of fine-grained semantic features of the code segments can improve the performance of malware family classification.

**Table 7:** Impact of different features description on classification performance.

Image type	Feature	Accuracy	Precision	Recall	F-measure	Time (ms)
bytecode	structural	0.9201	0.9212	0.9224	0.9218	30
opcode	semantic	0.9617	0.9534	0.9591	0.9562	38
bytecode+ opcode	structural+ semantic	0.9905	0.9871	0.9833	0.9852	42

**Figure 9:**
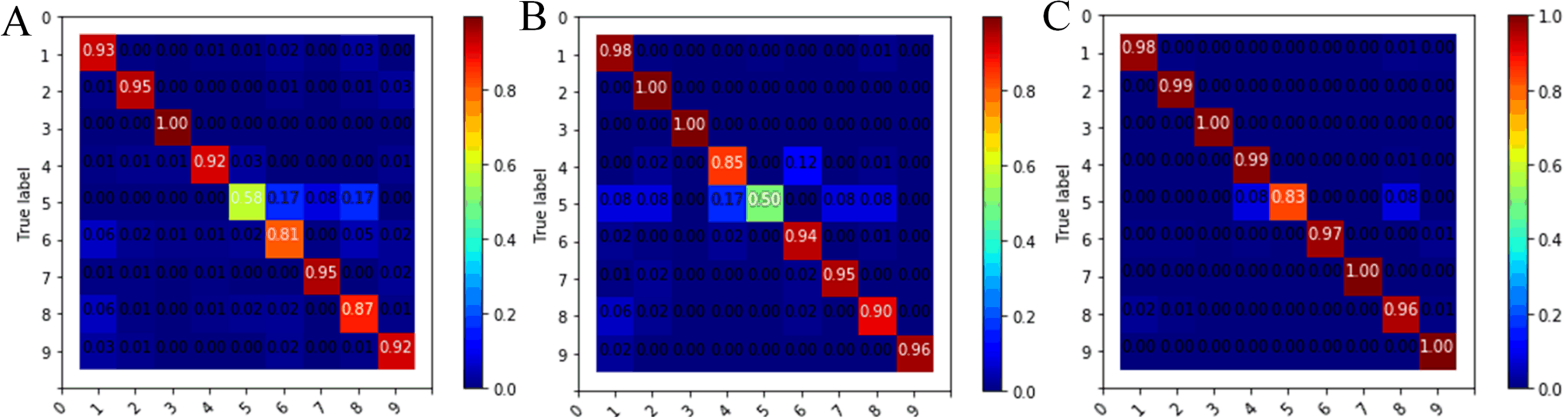
Confusion matrix of nine families for different feature descriptions. (A) Bytecode image feature. (B) Opcode image feature. (C) Bytecode+Opcode image feature.

The confusion matrix and classification accuracy of various malware families based on malware images of 64 × 64 is shown in Fig. 9. The sample number of Simda family was only 42 and the effective classification information of this category was insufficient, so the classification accuracy of this family was low in our experiment. However, the accuracy of the Simda family was still over 83%, which was improved by nearly half compared to using individual features. At the same time, we saw that the Obfuscator.ACY family (family ID: 8) achieved a classification accuracy rate of 96%, which was 9% higher than the single bytecode feature, indicating that our model can also achieve the ideal classification effect for confused malware. The classification accuracy of other families has also been improved to different degrees.

### Comparison with other works

To validate the advantages and efficiency of our proposed method, we compared our model with five advanced malware classification models. These models respectively adopt image feature extraction schemes based on malware grayscale image similar to bytecode image (Nataraj et al., 2011a; Luo & Lo, 2017; Cui et al., 2019; Venkatraman, Alazab & Vinayakumar, 2019) and text feature extraction scheme (Santos et al., 2013). As shown in Table 8, in terms of bytecode images, the accuracy of traditional image feature extraction technology such as Gist and local binary pattern (LBP) only reaches about 90%, while the accuracy of using CNN to classify malware images exceeds 95%, and the F-measure is also higher. These methods feed the image directly into the CNN network without additional feature extraction. Therefore, CNN is more suitable for the scenario of malware image classification than the traditional image feature extraction technology. The classification accuracy of n-gram text features is also about 90%, which are similar to the classification effect of bytecode images based on traditional image feature extraction technology. The double image feature fusion method proposed in this paper comprehensively considers the code segment n-gram feature and bytecode image of the malware, which has a higher classification accuracy (99.05%) and F-measure (98.52%) than other methods. The result shows that the fusion of the overall structural feature and the fine-grained feature of the opcode is effective for determining the homology of the malware family.

**Table 8:** Proposed model compared with other methods.

Method	Accuracy	Precision	Recall	F-measure
Gist+KNN (image)	0.8897	0.9150	0.9122	0.9081
LBP+KNN (image)	0.9110	0.9198	0.9563	0.9137
CNN (image)	0.9760	0.9310	0.8871	0.9085
Hybrid of CNN and GRU (image)	0.9651	0.9517	0.9439	0.9478
N-gram + KNN (text)	0.8908	0.8891	0.9197	0.9119
Proposed method	0.9905	0.9871	0.9833	0.9852

## Conclusions

We proposed a novel family classification method based on image visualization and feature fusion for homology determination of malware variants. We converted the malware executable into two feature representations of bytecode image and opcode image. The bytecode image represented the global structural features of the malware file, which was converted from binary file to grayscale image. Opcode images represented the local fine-grained features of malware code segments. The construction methods included key opcode extraction, *n*-gram feature extraction, opcode sequence vector and opcode image generation. We built a malware image classification model of dual-channel CNN using these construction methods, which fused the double image features and obtain homology determination results. We adopted the real dataset (Big 2015) from Microsoft Malware Classification Challenge to train and evaluate the model. Our results showed that the model had a 99.05% accuracy. Our proposed model improves the classification accuracy by 9%, particularly with confusing malware families, compared with using a single image feature. Compared with other major methods, our method outperforms others in the homology determination of the malware variants from different families.

## Supplemental Information

10.7717/peerj-cs.494/supp-1 Supplemental Information 1 The program python code and related intermediate files.

10.7717/peerj-cs.494/supp-2 Supplemental Information 2 10868 generated opcode images.

10.7717/peerj-cs.494/supp-3 Supplemental Information 3 10868 generated bytecode images.

## Additional Information and Declarations

### Data Availability

The following information was supplied regarding data availability: Data and code are available in the Supplemental Files.
