# Supplementary figures and images for: Malware homology determination using visualized images and feature fusion (part 2 of 4)
Source: PeerJ Comput Sci. 2021 Apr 15;7:e494. doi: 10.7717/peerj-cs.494 (PMC8056249; doi:10.7717/peerj-cs.494)

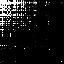

Supplement: Supplemental Information 2 [file peerj-cs-07-494-s002.zip › opcode_image/0fHVZKeTE6iRb1PIQ4au.jpg]

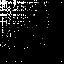

Supplement: Supplemental Information 2 [file peerj-cs-07-494-s002.zip › opcode_image/0FKerJl18xOc3jdoyg4A.jpg]

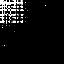

Supplement: Supplemental Information 2 [file peerj-cs-07-494-s002.zip › opcode_image/0FOXjzmnD9CUMVcSlEqh.jpg]

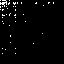

Supplement: Supplemental Information 2 [file peerj-cs-07-494-s002.zip › opcode_image/0Fu9oETtMW4zlg1ZrUy6.jpg]

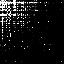

Supplement: Supplemental Information 2 [file peerj-cs-07-494-s002.zip › opcode_image/0fvnGU7dkbr8iEhZuMcP.jpg]

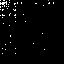

Supplement: Supplemental Information 2 [file peerj-cs-07-494-s002.zip › opcode_image/0fxgjYEClPL1BDbcshzJ.jpg]

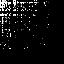

Supplement: Supplemental Information 2 [file peerj-cs-07-494-s002.zip › opcode_image/0G2RV1chBlIbkt6JqA5Q.jpg]

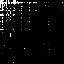

Supplement: Supplemental Information 2 [file peerj-cs-07-494-s002.zip › opcode_image/0G4hwobLuAzvl1PWYfmd.jpg]

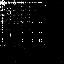

Supplement: Supplemental Information 2 [file peerj-cs-07-494-s002.zip › opcode_image/0GbMkYlNyt72OzBjIcVh.jpg]

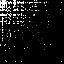

Supplement: Supplemental Information 2 [file peerj-cs-07-494-s002.zip › opcode_image/0gCmlyxw2UJvX7SNOGqu.jpg]

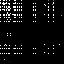

Supplement: Supplemental Information 2 [file peerj-cs-07-494-s002.zip › opcode_image/0gcZkSFr7VnEmLPbTxUe.jpg]

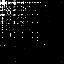

Supplement: Supplemental Information 2 [file peerj-cs-07-494-s002.zip › opcode_image/0gDsIvrylX5fPbG7cSBn.jpg]

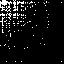

Supplement: Supplemental Information 2 [file peerj-cs-07-494-s002.zip › opcode_image/0gHs6DEouiCPAcmWFrTX.jpg]

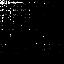

Supplement: Supplemental Information 2 [file peerj-cs-07-494-s002.zip › opcode_image/0giIqhw6e4mrHYzKFl8T.jpg]

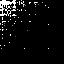

Supplement: Supplemental Information 2 [file peerj-cs-07-494-s002.zip › opcode_image/0gkj92oIleU4SYiCWpaM.jpg]

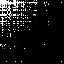

Supplement: Supplemental Information 2 [file peerj-cs-07-494-s002.zip › opcode_image/0GKp9ZJclxTABMunIOD2.jpg]

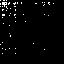

Supplement: Supplemental Information 2 [file peerj-cs-07-494-s002.zip › opcode_image/0GKzFQ81IYXqUWkmfv26.jpg]

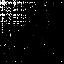

Supplement: Supplemental Information 2 [file peerj-cs-07-494-s002.zip › opcode_image/0gL3h5G6CszBV7RSinjJ.jpg]

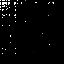

Supplement: Supplemental Information 2 [file peerj-cs-07-494-s002.zip › opcode_image/0glscKoNakWL84EpunPe.jpg]

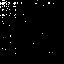

Supplement: Supplemental Information 2 [file peerj-cs-07-494-s002.zip › opcode_image/0gSm7QZu5x6MBvVzUncH.jpg]

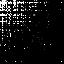

Supplement: Supplemental Information 2 [file peerj-cs-07-494-s002.zip › opcode_image/0Gu4misTcKynQD2Ol1Jx.jpg]

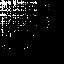

Supplement: Supplemental Information 2 [file peerj-cs-07-494-s002.zip › opcode_image/0GUIi7xAlODwZ4YBenNM.jpg]

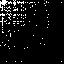

Supplement: Supplemental Information 2 [file peerj-cs-07-494-s002.zip › opcode_image/0gUpzkLVT73PCXx5WFRI.jpg]

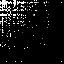

Supplement: Supplemental Information 2 [file peerj-cs-07-494-s002.zip › opcode_image/0GuYe4J7oLwQ82xr3pWS.jpg]

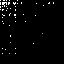

Supplement: Supplemental Information 2 [file peerj-cs-07-494-s002.zip › opcode_image/0GVcTdBQXWUJ2t7vjphN.jpg]

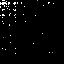

Supplement: Supplemental Information 2 [file peerj-cs-07-494-s002.zip › opcode_image/0GvtWEPUBfDAcMbiYVSR.jpg]

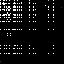

Supplement: Supplemental Information 2 [file peerj-cs-07-494-s002.zip › opcode_image/0gWUIudhwovMYb3NSnZA.jpg]

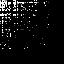

Supplement: Supplemental Information 2 [file peerj-cs-07-494-s002.zip › opcode_image/0gxJ1YmwFUvnOzoM8N53.jpg]

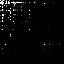

Supplement: Supplemental Information 2 [file peerj-cs-07-494-s002.zip › opcode_image/0H5OFklQm7wAPfE1qaJY.jpg]

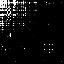

Supplement: Supplemental Information 2 [file peerj-cs-07-494-s002.zip › opcode_image/0H63jydvIahOVqgx5Kfo.jpg]

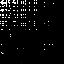

Supplement: Supplemental Information 2 [file peerj-cs-07-494-s002.zip › opcode_image/0hAlkjTR1Q6PewMczavb.jpg]

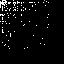

Supplement: Supplemental Information 2 [file peerj-cs-07-494-s002.zip › opcode_image/0hBIiRpkMZtoYj3lcDLa.jpg]

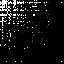

Supplement: Supplemental Information 2 [file peerj-cs-07-494-s002.zip › opcode_image/0HcZRmLi9VTpuQJCoXny.jpg]

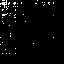

Supplement: Supplemental Information 2 [file peerj-cs-07-494-s002.zip › opcode_image/0hH3JB2wM79lYdsyuK5N.jpg]

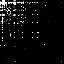

Supplement: Supplemental Information 2 [file peerj-cs-07-494-s002.zip › opcode_image/0HICT7RtjaVQzcNOeMgS.jpg]

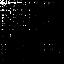

Supplement: Supplemental Information 2 [file peerj-cs-07-494-s002.zip › opcode_image/0HKFs3AXTt1IrOl52eVu.jpg]

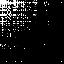

Supplement: Supplemental Information 2 [file peerj-cs-07-494-s002.zip › opcode_image/0HKM38fmCR5DrxoIkBnQ.jpg]

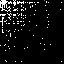

Supplement: Supplemental Information 2 [file peerj-cs-07-494-s002.zip › opcode_image/0Hlm4XgE1cQhC6BkMays.jpg]

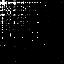

Supplement: Supplemental Information 2 [file peerj-cs-07-494-s002.zip › opcode_image/0Hn2ojct97Wp1TbdNM4Q.jpg]

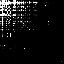

Supplement: Supplemental Information 2 [file peerj-cs-07-494-s002.zip › opcode_image/0Hrfce4X5YGESJPjl9uL.jpg]

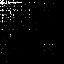

Supplement: Supplemental Information 2 [file peerj-cs-07-494-s002.zip › opcode_image/0HVAnMrp1LjKDmuoOJFY.jpg]

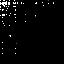

Supplement: Supplemental Information 2 [file peerj-cs-07-494-s002.zip › opcode_image/0hWRb28Umdgj7xcXOwtC.jpg]

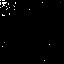

Supplement: Supplemental Information 2 [file peerj-cs-07-494-s002.zip › opcode_image/0hZEqJ5eMVjU21HAG7Ii.jpg]

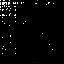

Supplement: Supplemental Information 2 [file peerj-cs-07-494-s002.zip › opcode_image/0hZqVRKkw7GfMdpalLiN.jpg]

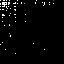

Supplement: Supplemental Information 2 [file peerj-cs-07-494-s002.zip › opcode_image/0i4ENysvVrgFnbaHUuJK.jpg]

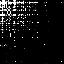

Supplement: Supplemental Information 2 [file peerj-cs-07-494-s002.zip › opcode_image/0i4FNJPQ8GuB3WU56LTS.jpg]

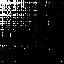

Supplement: Supplemental Information 2 [file peerj-cs-07-494-s002.zip › opcode_image/0I4ZVvngsAatm8fzD3pk.jpg]

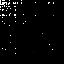

Supplement: Supplemental Information 2 [file peerj-cs-07-494-s002.zip › opcode_image/0iABvIkp3WHfgrJ79ymq.jpg]

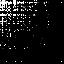

Supplement: Supplemental Information 2 [file peerj-cs-07-494-s002.zip › opcode_image/0IAlcuEiP9G6epb71Oom.jpg]

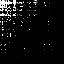

Supplement: Supplemental Information 2 [file peerj-cs-07-494-s002.zip › opcode_image/0iBaz3krsQ8HuA7cGDSt.jpg]

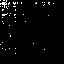

Supplement: Supplemental Information 2 [file peerj-cs-07-494-s002.zip › opcode_image/0icJrNnmPvDqVQkC3we1.jpg]

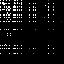

Supplement: Supplemental Information 2 [file peerj-cs-07-494-s002.zip › opcode_image/0IelgX5H2s14KutkEyNU.jpg]

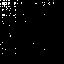

Supplement: Supplemental Information 2 [file peerj-cs-07-494-s002.zip › opcode_image/0IMUKlZs1Sm8LpGRkWhT.jpg]

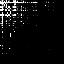

Supplement: Supplemental Information 2 [file peerj-cs-07-494-s002.zip › opcode_image/0iS3pwlgJco8XORD4TLq.jpg]

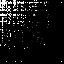

Supplement: Supplemental Information 2 [file peerj-cs-07-494-s002.zip › opcode_image/0isdESDMzq2K8T6FLPcC.jpg]

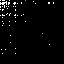

Supplement: Supplemental Information 2 [file peerj-cs-07-494-s002.zip › opcode_image/0itbI5mjJF28ocTkrUf9.jpg]

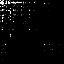

Supplement: Supplemental Information 2 [file peerj-cs-07-494-s002.zip › opcode_image/0ItXlAUOhK8ZYdDf7HW4.jpg]

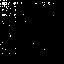

Supplement: Supplemental Information 2 [file peerj-cs-07-494-s002.zip › opcode_image/0IyaidUKRqnt2PDfOzHT.jpg]

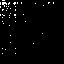

Supplement: Supplemental Information 2 [file peerj-cs-07-494-s002.zip › opcode_image/0IYZltU7uMpaco85PfKr.jpg]

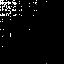

Supplement: Supplemental Information 2 [file peerj-cs-07-494-s002.zip › opcode_image/0iZTHuQ5KMb4RtAlrz6D.jpg]

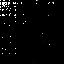

Supplement: Supplemental Information 2 [file peerj-cs-07-494-s002.zip › opcode_image/0J2pOclDKjadkL57eroz.jpg]

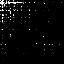

Supplement: Supplemental Information 2 [file peerj-cs-07-494-s002.zip › opcode_image/0J61YGoWjV25TzxeSluf.jpg]

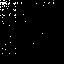

Supplement: Supplemental Information 2 [file peerj-cs-07-494-s002.zip › opcode_image/0jAopX629OwEH8WPkzVU.jpg]

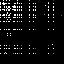

Supplement: Supplemental Information 2 [file peerj-cs-07-494-s002.zip › opcode_image/0JAx9gzbC54Q61XBrqc7.jpg]

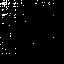

Supplement: Supplemental Information 2 [file peerj-cs-07-494-s002.zip › opcode_image/0JAzwGUKORhFQWr3o1dN.jpg]

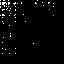

Supplement: Supplemental Information 2 [file peerj-cs-07-494-s002.zip › opcode_image/0JBNEWmdi7GptrK5qYD9.jpg]

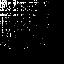

Supplement: Supplemental Information 2 [file peerj-cs-07-494-s002.zip › opcode_image/0JECiqrVNR1dgj67pZue.jpg]

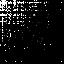

Supplement: Supplemental Information 2 [file peerj-cs-07-494-s002.zip › opcode_image/0JfwyrEcBqaRzN9TgFMh.jpg]

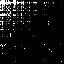

Supplement: Supplemental Information 2 [file peerj-cs-07-494-s002.zip › opcode_image/0jkmvR43UQ9yKxqXei61.jpg]

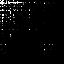

Supplement: Supplemental Information 2 [file peerj-cs-07-494-s002.zip › opcode_image/0jKSsqXVHNucByZ9l6Ao.jpg]

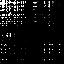

Supplement: Supplemental Information 2 [file peerj-cs-07-494-s002.zip › opcode_image/0JnvoeflBWwIcQa5GEPK.jpg]

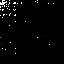

Supplement: Supplemental Information 2 [file peerj-cs-07-494-s002.zip › opcode_image/0JPAX13cjxewaTh6tRCi.jpg]

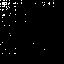

Supplement: Supplemental Information 2 [file peerj-cs-07-494-s002.zip › opcode_image/0K4sTCLtrIJ5SinQbe7u.jpg]

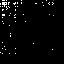

Supplement: Supplemental Information 2 [file peerj-cs-07-494-s002.zip › opcode_image/0K6yBUcTw3qjtNo4ZQpY.jpg]

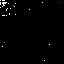

Supplement: Supplemental Information 2 [file peerj-cs-07-494-s002.zip › opcode_image/0KgE6ksUeytoHfl2cT4r.jpg]

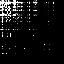

Supplement: Supplemental Information 2 [file peerj-cs-07-494-s002.zip › opcode_image/0KigmP9TLwJXNGz26tfO.jpg]

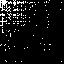

Supplement: Supplemental Information 2 [file peerj-cs-07-494-s002.zip › opcode_image/0KLUAMqmPJhOwaYrbSCE.jpg]

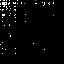

Supplement: Supplemental Information 2 [file peerj-cs-07-494-s002.zip › opcode_image/0KyDiQb1whgaSrmlx58J.jpg]

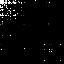

Supplement: Supplemental Information 2 [file peerj-cs-07-494-s002.zip › opcode_image/0KZFcsOYR4MdPJf6VvGS.jpg]

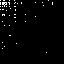

Supplement: Supplemental Information 2 [file peerj-cs-07-494-s002.zip › opcode_image/0kzRDUmBLHGd4YPj7hO6.jpg]

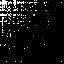

Supplement: Supplemental Information 2 [file peerj-cs-07-494-s002.zip › opcode_image/0l5IobyKpuqcwO4NxfgD.jpg]

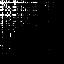

Supplement: Supplemental Information 2 [file peerj-cs-07-494-s002.zip › opcode_image/0l6fhCty3aSLDOgAjYQi.jpg]

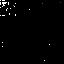

Supplement: Supplemental Information 2 [file peerj-cs-07-494-s002.zip › opcode_image/0LAXajqhQy7po16dw8Tx.jpg]

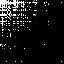

Supplement: Supplemental Information 2 [file peerj-cs-07-494-s002.zip › opcode_image/0LQSi5wnRZ3muIs6Mx9E.jpg]

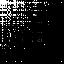

Supplement: Supplemental Information 2 [file peerj-cs-07-494-s002.zip › opcode_image/0LVqvlHF8PuepodIiBUb.jpg]

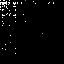

Supplement: Supplemental Information 2 [file peerj-cs-07-494-s002.zip › opcode_image/0LZkc7qeS39TUtVHuJB1.jpg]

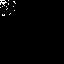

Supplement: Supplemental Information 2 [file peerj-cs-07-494-s002.zip › opcode_image/0M7aSiE9csDzkmfKheVt.jpg]

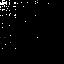

Supplement: Supplemental Information 2 [file peerj-cs-07-494-s002.zip › opcode_image/0m94tRnhgpsAUuY1L8KC.jpg]

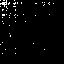

Supplement: Supplemental Information 2 [file peerj-cs-07-494-s002.zip › opcode_image/0mcWyK6unLRGV8Hfr97Y.jpg]

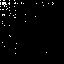

Supplement: Supplemental Information 2 [file peerj-cs-07-494-s002.zip › opcode_image/0meUjiuJvODcf3k9z4Iy.jpg]

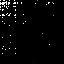

Supplement: Supplemental Information 2 [file peerj-cs-07-494-s002.zip › opcode_image/0mfwTlekXE1poYAnqMRO.jpg]

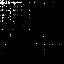

Supplement: Supplemental Information 2 [file peerj-cs-07-494-s002.zip › opcode_image/0mgFnqeLAMr7jthUYRTv.jpg]

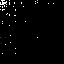

Supplement: Supplemental Information 2 [file peerj-cs-07-494-s002.zip › opcode_image/0mlhuKGpCc6OB4zwrbLy.jpg]

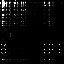

Supplement: Supplemental Information 2 [file peerj-cs-07-494-s002.zip › opcode_image/0MmZ8j5pn2R3VG9wlxYi.jpg]

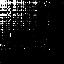

Supplement: Supplemental Information 2 [file peerj-cs-07-494-s002.zip › opcode_image/0MOorvEIRmZGhqQdc3TA.jpg]

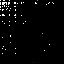

Supplement: Supplemental Information 2 [file peerj-cs-07-494-s002.zip › opcode_image/0MpJYhdbf8T7InoqcXr1.jpg]

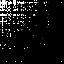

Supplement: Supplemental Information 2 [file peerj-cs-07-494-s002.zip › opcode_image/0MPV9Y8WNcFoyRZqQ76G.jpg]

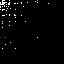

Supplement: Supplemental Information 2 [file peerj-cs-07-494-s002.zip › opcode_image/0MQD6mnoy4l3zV8WPRYe.jpg]

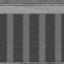

Supplement: Supplemental Information 3 [file peerj-cs-07-494-s003.zip › 02zcUmKV16Lya5xqnPGB.bytes.jpg]

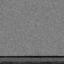

Supplement: Supplemental Information 3 [file peerj-cs-07-494-s003.zip › 03nJaQV6K2ObICUmyWoR.bytes.jpg]
