# Supplementary figures and images for: Malware homology determination using visualized images and feature fusion (part 1 of 4)
Source: PeerJ Comput Sci. 2021 Apr 15;7:e494. doi: 10.7717/peerj-cs.494 (PMC8056249; doi:10.7717/peerj-cs.494)

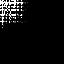

Supplement: Supplemental Information 2 [file peerj-cs-07-494-s002.zip › opcode_image/01azqd4InC7m9JpocGv5.jpg]

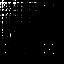

Supplement: Supplemental Information 2 [file peerj-cs-07-494-s002.zip › opcode_image/01IsoiSMh5gxyDYTl4CB.jpg]

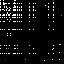

Supplement: Supplemental Information 2 [file peerj-cs-07-494-s002.zip › opcode_image/01jsnpXSAlgw6aPeDxrU.jpg]

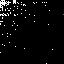

Supplement: Supplemental Information 2 [file peerj-cs-07-494-s002.zip › opcode_image/01kcPWA9K2BOxQeS5Rju.jpg]

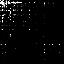

Supplement: Supplemental Information 2 [file peerj-cs-07-494-s002.zip › opcode_image/01SuzwMJEIXsK7A8dQbl.jpg]

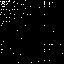

Supplement: Supplemental Information 2 [file peerj-cs-07-494-s002.zip › opcode_image/02IOCvYEy8mjiuAQHax3.jpg]

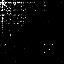

Supplement: Supplemental Information 2 [file peerj-cs-07-494-s002.zip › opcode_image/02JqQ7H3yEoD8viYWlmS.jpg]

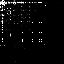

Supplement: Supplemental Information 2 [file peerj-cs-07-494-s002.zip › opcode_image/02K5GMYITj7bBoAisEmD.jpg]

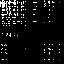

Supplement: Supplemental Information 2 [file peerj-cs-07-494-s002.zip › opcode_image/02mlBLHZTDFXGa7Nt6cr.jpg]

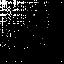

Supplement: Supplemental Information 2 [file peerj-cs-07-494-s002.zip › opcode_image/02MRILoE6rNhmt7FUi45.jpg]

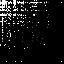

Supplement: Supplemental Information 2 [file peerj-cs-07-494-s002.zip › opcode_image/02zcUmKV16Lya5xqnPGB.jpg]

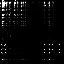

Supplement: Supplemental Information 2 [file peerj-cs-07-494-s002.zip › opcode_image/03nJaQV6K2ObICUmyWoR.jpg]

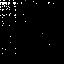

Supplement: Supplemental Information 2 [file peerj-cs-07-494-s002.zip › opcode_image/04BfoQRA6XEshiNuI7pF.jpg]

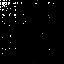

Supplement: Supplemental Information 2 [file peerj-cs-07-494-s002.zip › opcode_image/04cvLCVPqBMs6yn5xGlE.jpg]

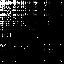

Supplement: Supplemental Information 2 [file peerj-cs-07-494-s002.zip › opcode_image/04EjIdbPV5e1XroFOpiN.jpg]

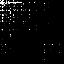

Supplement: Supplemental Information 2 [file peerj-cs-07-494-s002.zip › opcode_image/04hSzLv5s2TDYPlcgpHB.jpg]

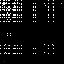

Supplement: Supplemental Information 2 [file peerj-cs-07-494-s002.zip › opcode_image/04mcPSei852tgIKUwTJr.jpg]

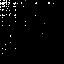

Supplement: Supplemental Information 2 [file peerj-cs-07-494-s002.zip › opcode_image/04QzZ3DVdPsEp9elLR65.jpg]

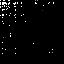

Supplement: Supplemental Information 2 [file peerj-cs-07-494-s002.zip › opcode_image/04sJnMaORYc1SV5pKjrP.jpg]

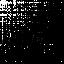

Supplement: Supplemental Information 2 [file peerj-cs-07-494-s002.zip › opcode_image/05aiMRw13bYWqZ8OHvjl.jpg]

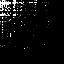

Supplement: Supplemental Information 2 [file peerj-cs-07-494-s002.zip › opcode_image/05EeG39MTRrI6VY21DPd.jpg]

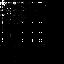

Supplement: Supplemental Information 2 [file peerj-cs-07-494-s002.zip › opcode_image/05IXcWGxvnkto4sq17zZ.jpg]

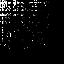

Supplement: Supplemental Information 2 [file peerj-cs-07-494-s002.zip › opcode_image/05Kps4iFw8mOLJZQrb1H.jpg]

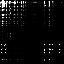

Supplement: Supplemental Information 2 [file peerj-cs-07-494-s002.zip › opcode_image/05LHG8fR3iPn6agIo9z7.jpg]

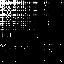

Supplement: Supplemental Information 2 [file peerj-cs-07-494-s002.zip › opcode_image/05rJTUWYAKNegBk2wE8X.jpg]

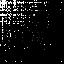

Supplement: Supplemental Information 2 [file peerj-cs-07-494-s002.zip › opcode_image/065EZhxgbLRSHsB87uIF.jpg]

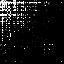

Supplement: Supplemental Information 2 [file peerj-cs-07-494-s002.zip › opcode_image/06aLOj8EUXMByS423sum.jpg]

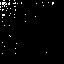

Supplement: Supplemental Information 2 [file peerj-cs-07-494-s002.zip › opcode_image/06arUi9q3wHS2C8RZxeB.jpg]

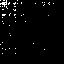

Supplement: Supplemental Information 2 [file peerj-cs-07-494-s002.zip › opcode_image/06KfrF7ltESna2ZHPVp5.jpg]

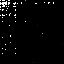

Supplement: Supplemental Information 2 [file peerj-cs-07-494-s002.zip › opcode_image/06osXqPUVM1HbvBGNncT.jpg]

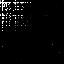

Supplement: Supplemental Information 2 [file peerj-cs-07-494-s002.zip › opcode_image/06QinlpeFIWj8qHc7Vys.jpg]

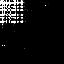

Supplement: Supplemental Information 2 [file peerj-cs-07-494-s002.zip › opcode_image/07ECKjDTyQLnabNoxrIH.jpg]

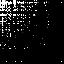

Supplement: Supplemental Information 2 [file peerj-cs-07-494-s002.zip › opcode_image/07iSOIG2urUvsMl9E5Rn.jpg]

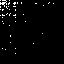

Supplement: Supplemental Information 2 [file peerj-cs-07-494-s002.zip › opcode_image/07nrG1cLKUPxjOlWMFiV.jpg]

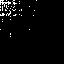

Supplement: Supplemental Information 2 [file peerj-cs-07-494-s002.zip › opcode_image/08BX5Slp2I1FraZWbc6j.jpg]

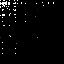

Supplement: Supplemental Information 2 [file peerj-cs-07-494-s002.zip › opcode_image/09bfacpUzuBN5W3S8KTo.jpg]

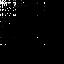

Supplement: Supplemental Information 2 [file peerj-cs-07-494-s002.zip › opcode_image/09CPNMYyQjSguFrE8UOf.jpg]

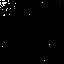

Supplement: Supplemental Information 2 [file peerj-cs-07-494-s002.zip › opcode_image/09LXtWxm1EbK5uVqcQS3.jpg]

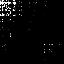

Supplement: Supplemental Information 2 [file peerj-cs-07-494-s002.zip › opcode_image/09sXMJUHwQWVanrhzAoT.jpg]

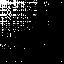

Supplement: Supplemental Information 2 [file peerj-cs-07-494-s002.zip › opcode_image/0A32eTdBKayjCWhZqDOQ.jpg]

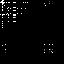

Supplement: Supplemental Information 2 [file peerj-cs-07-494-s002.zip › opcode_image/0ACDbR5M3ZhBJajygTuf.jpg]

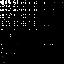

Supplement: Supplemental Information 2 [file peerj-cs-07-494-s002.zip › opcode_image/0AguvpOCcaf2myVDYFGb.jpg]

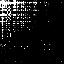

Supplement: Supplemental Information 2 [file peerj-cs-07-494-s002.zip › opcode_image/0akIgwhWHYm1dzsNqBFx.jpg]

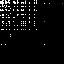

Supplement: Supplemental Information 2 [file peerj-cs-07-494-s002.zip › opcode_image/0aKlH1MRxLmv34QGhEJP.jpg]

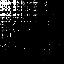

Supplement: Supplemental Information 2 [file peerj-cs-07-494-s002.zip › opcode_image/0AnoOZDNbPXIr2MRBSCJ.jpg]

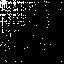

Supplement: Supplemental Information 2 [file peerj-cs-07-494-s002.zip › opcode_image/0ASH2csN7k8jZyoRaqtn.jpg]

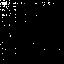

Supplement: Supplemental Information 2 [file peerj-cs-07-494-s002.zip › opcode_image/0aSTGBVRXeJhx5OcpsgC.jpg]

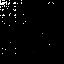

Supplement: Supplemental Information 2 [file peerj-cs-07-494-s002.zip › opcode_image/0aU7XWsr8RtN94jvo3lG.jpg]

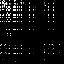

Supplement: Supplemental Information 2 [file peerj-cs-07-494-s002.zip › opcode_image/0AV6MPlrTWG4fYI7NBtQ.jpg]

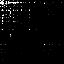

Supplement: Supplemental Information 2 [file peerj-cs-07-494-s002.zip › opcode_image/0aVNj3qFgEZI6Akf4Kuv.jpg]

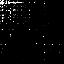

Supplement: Supplemental Information 2 [file peerj-cs-07-494-s002.zip › opcode_image/0aVxkvmflEizUBG2rMT4.jpg]

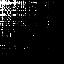

Supplement: Supplemental Information 2 [file peerj-cs-07-494-s002.zip › opcode_image/0AwWs42SUQ19mI7eDcTC.jpg]

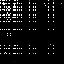

Supplement: Supplemental Information 2 [file peerj-cs-07-494-s002.zip › opcode_image/0B2RwKm6dq9fjUWDNIOa.jpg]

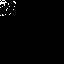

Supplement: Supplemental Information 2 [file peerj-cs-07-494-s002.zip › opcode_image/0b5LqcWix3J4fGIEhXQu.jpg]

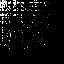

Supplement: Supplemental Information 2 [file peerj-cs-07-494-s002.zip › opcode_image/0BEsCP7NAUy8XmkenHWG.jpg]

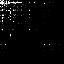

Supplement: Supplemental Information 2 [file peerj-cs-07-494-s002.zip › opcode_image/0BFIPv1rO83whtpMYyAs.jpg]

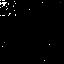

Supplement: Supplemental Information 2 [file peerj-cs-07-494-s002.zip › opcode_image/0BIdbVDEgmPwjYF4xzir.jpg]

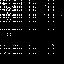

Supplement: Supplemental Information 2 [file peerj-cs-07-494-s002.zip › opcode_image/0bjN3Kgw5OATSreRmEdi.jpg]

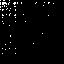

Supplement: Supplemental Information 2 [file peerj-cs-07-494-s002.zip › opcode_image/0BKcmNv4iGY2hsVSaXJ6.jpg]

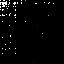

Supplement: Supplemental Information 2 [file peerj-cs-07-494-s002.zip › opcode_image/0BLbmzJRkjNynCgQIdtV.jpg]

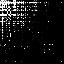

Supplement: Supplemental Information 2 [file peerj-cs-07-494-s002.zip › opcode_image/0bN6ODYWw2xeCQBn3tEg.jpg]

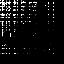

Supplement: Supplemental Information 2 [file peerj-cs-07-494-s002.zip › opcode_image/0BY2iPso3bEmudlUzpfq.jpg]

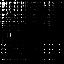

Supplement: Supplemental Information 2 [file peerj-cs-07-494-s002.zip › opcode_image/0BZQIJak6Pu2tyAXfrzR.jpg]

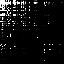

Supplement: Supplemental Information 2 [file peerj-cs-07-494-s002.zip › opcode_image/0C4aVbN58O1nAigFJt9z.jpg]

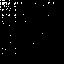

Supplement: Supplemental Information 2 [file peerj-cs-07-494-s002.zip › opcode_image/0cdnSIvN489sFUwYlrMQ.jpg]

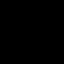

Supplement: Supplemental Information 2 [file peerj-cs-07-494-s002.zip › opcode_image/0cfGJLYgE6ROaZH7KT1h.jpg]

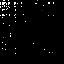

Supplement: Supplemental Information 2 [file peerj-cs-07-494-s002.zip › opcode_image/0cfIE39ihRNo2rkZOw5H.jpg]

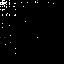

Supplement: Supplemental Information 2 [file peerj-cs-07-494-s002.zip › opcode_image/0cGWK6VvCkm7O2AxDjtw.jpg]

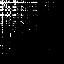

Supplement: Supplemental Information 2 [file peerj-cs-07-494-s002.zip › opcode_image/0cH8YeO15ZywEhPrJvmj.jpg]

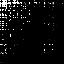

Supplement: Supplemental Information 2 [file peerj-cs-07-494-s002.zip › opcode_image/0co46B8IkPt2UN3HSaw7.jpg]

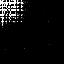

Supplement: Supplemental Information 2 [file peerj-cs-07-494-s002.zip › opcode_image/0CPaAXtyswrBq83D6VEg.jpg]

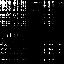

Supplement: Supplemental Information 2 [file peerj-cs-07-494-s002.zip › opcode_image/0Cq4wfhLrKBJiut1lYAZ.jpg]

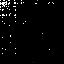

Supplement: Supplemental Information 2 [file peerj-cs-07-494-s002.zip › opcode_image/0csgzpwdL3FbZEJu6DjO.jpg]

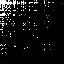

Supplement: Supplemental Information 2 [file peerj-cs-07-494-s002.zip › opcode_image/0cTu2bkefOAJqIhYUWFK.jpg]

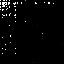

Supplement: Supplemental Information 2 [file peerj-cs-07-494-s002.zip › opcode_image/0CzL6rfwaTqGOu9eghBt.jpg]

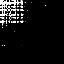

Supplement: Supplemental Information 2 [file peerj-cs-07-494-s002.zip › opcode_image/0czUXKSCiGY2j5mxLdWa.jpg]

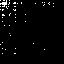

Supplement: Supplemental Information 2 [file peerj-cs-07-494-s002.zip › opcode_image/0D9IedmC1viTPugLRWX6.jpg]

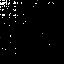

Supplement: Supplemental Information 2 [file peerj-cs-07-494-s002.zip › opcode_image/0daTri9PSkeEsVHu5Dhw.jpg]

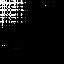

Supplement: Supplemental Information 2 [file peerj-cs-07-494-s002.zip › opcode_image/0dauMIK4ATfybzqUgNLc.jpg]

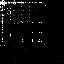

Supplement: Supplemental Information 2 [file peerj-cs-07-494-s002.zip › opcode_image/0DbLeKSoxu47wjqVHsi9.jpg]

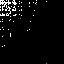

Supplement: Supplemental Information 2 [file peerj-cs-07-494-s002.zip › opcode_image/0df4cbsTBCn1VGW8lQRv.jpg]

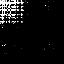

Supplement: Supplemental Information 2 [file peerj-cs-07-494-s002.zip › opcode_image/0dhL8Jvcswa7U1qHiDS5.jpg]

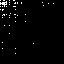

Supplement: Supplemental Information 2 [file peerj-cs-07-494-s002.zip › opcode_image/0Dk7Wd8MERu3b5rmQzCK.jpg]

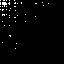

Supplement: Supplemental Information 2 [file peerj-cs-07-494-s002.zip › opcode_image/0dkuzUXLTEFwW71vP5bS.jpg]

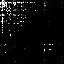

Supplement: Supplemental Information 2 [file peerj-cs-07-494-s002.zip › opcode_image/0DM3hS6Gg2QVKb1fZydv.jpg]

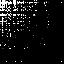

Supplement: Supplemental Information 2 [file peerj-cs-07-494-s002.zip › opcode_image/0dnTixlMYzDUpsvEVrGc.jpg]

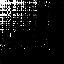

Supplement: Supplemental Information 2 [file peerj-cs-07-494-s002.zip › opcode_image/0DNVFKwYlcjO7bTfJ5p1.jpg]

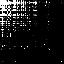

Supplement: Supplemental Information 2 [file peerj-cs-07-494-s002.zip › opcode_image/0DqUX5rkg3IbMY6BLGCE.jpg]

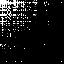

Supplement: Supplemental Information 2 [file peerj-cs-07-494-s002.zip › opcode_image/0DTp59Av1RLifoKlUdm7.jpg]

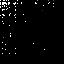

Supplement: Supplemental Information 2 [file peerj-cs-07-494-s002.zip › opcode_image/0DTs2PhZfCwEv7q8349K.jpg]

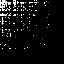

Supplement: Supplemental Information 2 [file peerj-cs-07-494-s002.zip › opcode_image/0EAdHtLDypMcwjTFJziC.jpg]

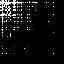

Supplement: Supplemental Information 2 [file peerj-cs-07-494-s002.zip › opcode_image/0eaNKwluUmkYdIvZ923c.jpg]

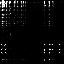

Supplement: Supplemental Information 2 [file peerj-cs-07-494-s002.zip › opcode_image/0EL7OGZKozbiNCVP61gk.jpg]

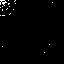

Supplement: Supplemental Information 2 [file peerj-cs-07-494-s002.zip › opcode_image/0eN9lyQfwmTVk7C2ZoYp.jpg]

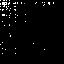

Supplement: Supplemental Information 2 [file peerj-cs-07-494-s002.zip › opcode_image/0Eo9qT6idXHDMebwmvPA.jpg]

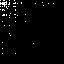

Supplement: Supplemental Information 2 [file peerj-cs-07-494-s002.zip › opcode_image/0evDQX7AVfC1ZTJEKltg.jpg]

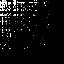

Supplement: Supplemental Information 2 [file peerj-cs-07-494-s002.zip › opcode_image/0F4qIHaR7xOrm19Set3o.jpg]

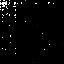

Supplement: Supplemental Information 2 [file peerj-cs-07-494-s002.zip › opcode_image/0FdOaDWrfBU6TqwCRYxA.jpg]

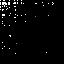

Supplement: Supplemental Information 2 [file peerj-cs-07-494-s002.zip › opcode_image/0fGuCWgTraQ6nEmLPN8q.jpg]

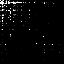

Supplement: Supplemental Information 2 [file peerj-cs-07-494-s002.zip › opcode_image/0fhnXI9ESr4jgWmkiaTe.jpg]
