# Supplementary figures and images for: Malware homology determination using visualized images and feature fusion (part 4 of 4)
Source: PeerJ Comput Sci. 2021 Apr 15;7:e494. doi: 10.7717/peerj-cs.494 (PMC8056249; doi:10.7717/peerj-cs.494)

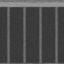

Supplement: Supplemental Information 3 [file peerj-cs-07-494-s003.zip › 0gHs6DEouiCPAcmWFrTX.bytes.jpg]

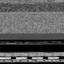

Supplement: Supplemental Information 3 [file peerj-cs-07-494-s003.zip › 0giIqhw6e4mrHYzKFl8T.bytes.jpg]

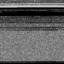

Supplement: Supplemental Information 3 [file peerj-cs-07-494-s003.zip › 0gkj92oIleU4SYiCWpaM.bytes.jpg]

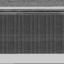

Supplement: Supplemental Information 3 [file peerj-cs-07-494-s003.zip › 0GKp9ZJclxTABMunIOD2.bytes.jpg]

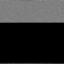

Supplement: Supplemental Information 3 [file peerj-cs-07-494-s003.zip › 0GKzFQ81IYXqUWkmfv26.bytes.jpg]

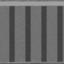

Supplement: Supplemental Information 3 [file peerj-cs-07-494-s003.zip › 0gL3h5G6CszBV7RSinjJ.bytes.jpg]

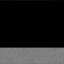

Supplement: Supplemental Information 3 [file peerj-cs-07-494-s003.zip › 0glscKoNakWL84EpunPe.bytes.jpg]

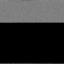

Supplement: Supplemental Information 3 [file peerj-cs-07-494-s003.zip › 0gSm7QZu5x6MBvVzUncH.bytes.jpg]

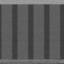

Supplement: Supplemental Information 3 [file peerj-cs-07-494-s003.zip › 0Gu4misTcKynQD2Ol1Jx.bytes.jpg]

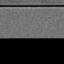

Supplement: Supplemental Information 3 [file peerj-cs-07-494-s003.zip › 0GUIi7xAlODwZ4YBenNM.bytes.jpg]

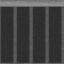

Supplement: Supplemental Information 3 [file peerj-cs-07-494-s003.zip › 0gUpzkLVT73PCXx5WFRI.bytes.jpg]

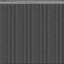

Supplement: Supplemental Information 3 [file peerj-cs-07-494-s003.zip › 0GuYe4J7oLwQ82xr3pWS.bytes.jpg]

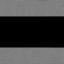

Supplement: Supplemental Information 3 [file peerj-cs-07-494-s003.zip › 0GVcTdBQXWUJ2t7vjphN.bytes.jpg]

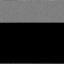

Supplement: Supplemental Information 3 [file peerj-cs-07-494-s003.zip › 0GvtWEPUBfDAcMbiYVSR.bytes.jpg]

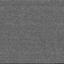

Supplement: Supplemental Information 3 [file peerj-cs-07-494-s003.zip › 0gWUIudhwovMYb3NSnZA.bytes.jpg]

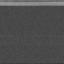

Supplement: Supplemental Information 3 [file peerj-cs-07-494-s003.zip › 0gxJ1YmwFUvnOzoM8N53.bytes.jpg]

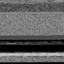

Supplement: Supplemental Information 3 [file peerj-cs-07-494-s003.zip › 0H5OFklQm7wAPfE1qaJY.bytes.jpg]

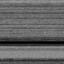

Supplement: Supplemental Information 3 [file peerj-cs-07-494-s003.zip › 0H63jydvIahOVqgx5Kfo.bytes.jpg]

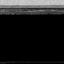

Supplement: Supplemental Information 3 [file peerj-cs-07-494-s003.zip › 0hAlkjTR1Q6PewMczavb.bytes.jpg]

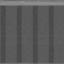

Supplement: Supplemental Information 3 [file peerj-cs-07-494-s003.zip › 0hBIiRpkMZtoYj3lcDLa.bytes.jpg]

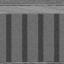

Supplement: Supplemental Information 3 [file peerj-cs-07-494-s003.zip › 0HcZRmLi9VTpuQJCoXny.bytes.jpg]

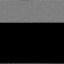

Supplement: Supplemental Information 3 [file peerj-cs-07-494-s003.zip › 0hH3JB2wM79lYdsyuK5N.bytes.jpg]

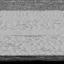

Supplement: Supplemental Information 3 [file peerj-cs-07-494-s003.zip › 0HICT7RtjaVQzcNOeMgS.bytes.jpg]

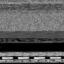

Supplement: Supplemental Information 3 [file peerj-cs-07-494-s003.zip › 0HKFs3AXTt1IrOl52eVu.bytes.jpg]

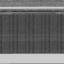

Supplement: Supplemental Information 3 [file peerj-cs-07-494-s003.zip › 0HKM38fmCR5DrxoIkBnQ.bytes.jpg]

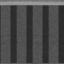

Supplement: Supplemental Information 3 [file peerj-cs-07-494-s003.zip › 0Hlm4XgE1cQhC6BkMays.bytes.jpg]

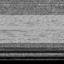

Supplement: Supplemental Information 3 [file peerj-cs-07-494-s003.zip › 0Hn2ojct97Wp1TbdNM4Q.bytes.jpg]

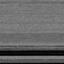

Supplement: Supplemental Information 3 [file peerj-cs-07-494-s003.zip › 0Hrfce4X5YGESJPjl9uL.bytes.jpg]

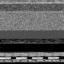

Supplement: Supplemental Information 3 [file peerj-cs-07-494-s003.zip › 0HVAnMrp1LjKDmuoOJFY.bytes.jpg]

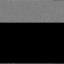

Supplement: Supplemental Information 3 [file peerj-cs-07-494-s003.zip › 0hWRb28Umdgj7xcXOwtC.bytes.jpg]

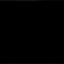

Supplement: Supplemental Information 3 [file peerj-cs-07-494-s003.zip › 0hZEqJ5eMVjU21HAG7Ii.bytes.jpg]

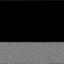

Supplement: Supplemental Information 3 [file peerj-cs-07-494-s003.zip › 0hZqVRKkw7GfMdpalLiN.bytes.jpg]

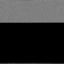

Supplement: Supplemental Information 3 [file peerj-cs-07-494-s003.zip › 0i4ENysvVrgFnbaHUuJK.bytes.jpg]

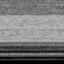

Supplement: Supplemental Information 3 [file peerj-cs-07-494-s003.zip › 0i4FNJPQ8GuB3WU56LTS.bytes.jpg]

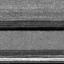

Supplement: Supplemental Information 3 [file peerj-cs-07-494-s003.zip › 0I4ZVvngsAatm8fzD3pk.bytes.jpg]

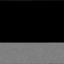

Supplement: Supplemental Information 3 [file peerj-cs-07-494-s003.zip › 0iABvIkp3WHfgrJ79ymq.bytes.jpg]

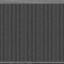

Supplement: Supplemental Information 3 [file peerj-cs-07-494-s003.zip › 0IAlcuEiP9G6epb71Oom.bytes.jpg]

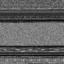

Supplement: Supplemental Information 3 [file peerj-cs-07-494-s003.zip › 0iBaz3krsQ8HuA7cGDSt.bytes.jpg]

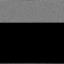

Supplement: Supplemental Information 3 [file peerj-cs-07-494-s003.zip › 0icJrNnmPvDqVQkC3we1.bytes.jpg]

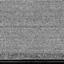

Supplement: Supplemental Information 3 [file peerj-cs-07-494-s003.zip › 0IelgX5H2s14KutkEyNU.bytes.jpg]

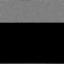

Supplement: Supplemental Information 3 [file peerj-cs-07-494-s003.zip › 0IMUKlZs1Sm8LpGRkWhT.bytes.jpg]

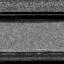

Supplement: Supplemental Information 3 [file peerj-cs-07-494-s003.zip › 0iS3pwlgJco8XORD4TLq.bytes.jpg]

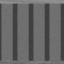

Supplement: Supplemental Information 3 [file peerj-cs-07-494-s003.zip › 0isdESDMzq2K8T6FLPcC.bytes.jpg]

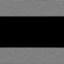

Supplement: Supplemental Information 3 [file peerj-cs-07-494-s003.zip › 0itbI5mjJF28ocTkrUf9.bytes.jpg]

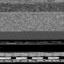

Supplement: Supplemental Information 3 [file peerj-cs-07-494-s003.zip › 0ItXlAUOhK8ZYdDf7HW4.bytes.jpg]

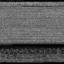

Supplement: Supplemental Information 3 [file peerj-cs-07-494-s003.zip › 0Iv6U2hbcP1xeBitW5Oo.bytes.jpg]

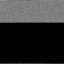

Supplement: Supplemental Information 3 [file peerj-cs-07-494-s003.zip › 0IyaidUKRqnt2PDfOzHT.bytes.jpg]

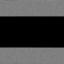

Supplement: Supplemental Information 3 [file peerj-cs-07-494-s003.zip › 0IYZltU7uMpaco85PfKr.bytes.jpg]

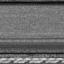

Supplement: Supplemental Information 3 [file peerj-cs-07-494-s003.zip › 0iZTHuQ5KMb4RtAlrz6D.bytes.jpg]

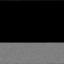

Supplement: Supplemental Information 3 [file peerj-cs-07-494-s003.zip › 0J2pOclDKjadkL57eroz.bytes.jpg]

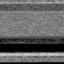

Supplement: Supplemental Information 3 [file peerj-cs-07-494-s003.zip › 0J61YGoWjV25TzxeSluf.bytes.jpg]

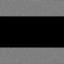

Supplement: Supplemental Information 3 [file peerj-cs-07-494-s003.zip › 0jAopX629OwEH8WPkzVU.bytes.jpg]

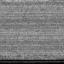

Supplement: Supplemental Information 3 [file peerj-cs-07-494-s003.zip › 0JAx9gzbC54Q61XBrqc7.bytes.jpg]

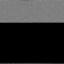

Supplement: Supplemental Information 3 [file peerj-cs-07-494-s003.zip › 0JAzwGUKORhFQWr3o1dN.bytes.jpg]

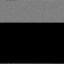

Supplement: Supplemental Information 3 [file peerj-cs-07-494-s003.zip › 0JBNEWmdi7GptrK5qYD9.bytes.jpg]

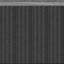

Supplement: Supplemental Information 3 [file peerj-cs-07-494-s003.zip › 0JECiqrVNR1dgj67pZue.bytes.jpg]

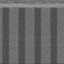

Supplement: Supplemental Information 3 [file peerj-cs-07-494-s003.zip › 0JfwyrEcBqaRzN9TgFMh.bytes.jpg]

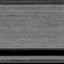

Supplement: Supplemental Information 3 [file peerj-cs-07-494-s003.zip › 0jkmvR43UQ9yKxqXei61.bytes.jpg]

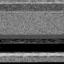

Supplement: Supplemental Information 3 [file peerj-cs-07-494-s003.zip › 0jKSsqXVHNucByZ9l6Ao.bytes.jpg]

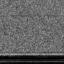

Supplement: Supplemental Information 3 [file peerj-cs-07-494-s003.zip › 0JnvoeflBWwIcQa5GEPK.bytes.jpg]

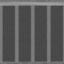

Supplement: Supplemental Information 3 [file peerj-cs-07-494-s003.zip › 0JOb8TyN6VBGCrjAkzfP.bytes.jpg]

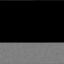

Supplement: Supplemental Information 3 [file peerj-cs-07-494-s003.zip › 0JPAX13cjxewaTh6tRCi.bytes.jpg]

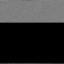

Supplement: Supplemental Information 3 [file peerj-cs-07-494-s003.zip › 0K4sTCLtrIJ5SinQbe7u.bytes.jpg]

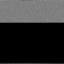

Supplement: Supplemental Information 3 [file peerj-cs-07-494-s003.zip › 0K6yBUcTw3qjtNo4ZQpY.bytes.jpg]

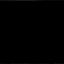

Supplement: Supplemental Information 3 [file peerj-cs-07-494-s003.zip › 0KgE6ksUeytoHfl2cT4r.bytes.jpg]

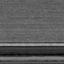

Supplement: Supplemental Information 3 [file peerj-cs-07-494-s003.zip › 0KigmP9TLwJXNGz26tfO.bytes.jpg]

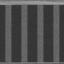

Supplement: Supplemental Information 3 [file peerj-cs-07-494-s003.zip › 0KLUAMqmPJhOwaYrbSCE.bytes.jpg]

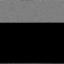

Supplement: Supplemental Information 3 [file peerj-cs-07-494-s003.zip › 0KyDiQb1whgaSrmlx58J.bytes.jpg]

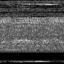

Supplement: Supplemental Information 3 [file peerj-cs-07-494-s003.zip › 0KZFcsOYR4MdPJf6VvGS.bytes.jpg]

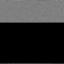

Supplement: Supplemental Information 3 [file peerj-cs-07-494-s003.zip › 0kzRDUmBLHGd4YPj7hO6.bytes.jpg]

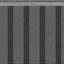

Supplement: Supplemental Information 3 [file peerj-cs-07-494-s003.zip › 0l5IobyKpuqcwO4NxfgD.bytes.jpg]

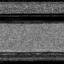

Supplement: Supplemental Information 3 [file peerj-cs-07-494-s003.zip › 0l6fhCty3aSLDOgAjYQi.bytes.jpg]

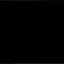

Supplement: Supplemental Information 3 [file peerj-cs-07-494-s003.zip › 0LAXajqhQy7po16dw8Tx.bytes.jpg]

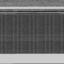

Supplement: Supplemental Information 3 [file peerj-cs-07-494-s003.zip › 0LQSi5wnRZ3muIs6Mx9E.bytes.jpg]

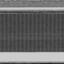

Supplement: Supplemental Information 3 [file peerj-cs-07-494-s003.zip › 0LVqvlHF8PuepodIiBUb.bytes.jpg]

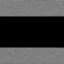

Supplement: Supplemental Information 3 [file peerj-cs-07-494-s003.zip › 0LZkc7qeS39TUtVHuJB1.bytes.jpg]

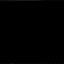

Supplement: Supplemental Information 3 [file peerj-cs-07-494-s003.zip › 0M7aSiE9csDzkmfKheVt.bytes.jpg]

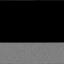

Supplement: Supplemental Information 3 [file peerj-cs-07-494-s003.zip › 0m94tRnhgpsAUuY1L8KC.bytes.jpg]

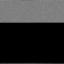

Supplement: Supplemental Information 3 [file peerj-cs-07-494-s003.zip › 0mcWyK6unLRGV8Hfr97Y.bytes.jpg]

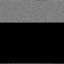

Supplement: Supplemental Information 3 [file peerj-cs-07-494-s003.zip › 0meUjiuJvODcf3k9z4Iy.bytes.jpg]

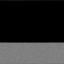

Supplement: Supplemental Information 3 [file peerj-cs-07-494-s003.zip › 0mfwTlekXE1poYAnqMRO.bytes.jpg]

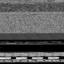

Supplement: Supplemental Information 3 [file peerj-cs-07-494-s003.zip › 0mgFnqeLAMr7jthUYRTv.bytes.jpg]

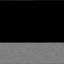

Supplement: Supplemental Information 3 [file peerj-cs-07-494-s003.zip › 0mlhuKGpCc6OB4zwrbLy.bytes.jpg]

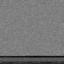

Supplement: Supplemental Information 3 [file peerj-cs-07-494-s003.zip › 0MmZ8j5pn2R3VG9wlxYi.bytes.jpg]

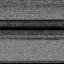

Supplement: Supplemental Information 3 [file peerj-cs-07-494-s003.zip › 0MOorvEIRmZGhqQdc3TA.bytes.jpg]

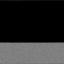

Supplement: Supplemental Information 3 [file peerj-cs-07-494-s003.zip › 0MpJYhdbf8T7InoqcXr1.bytes.jpg]

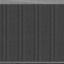

Supplement: Supplemental Information 3 [file peerj-cs-07-494-s003.zip › 0MPV9Y8WNcFoyRZqQ76G.bytes.jpg]

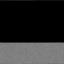

Supplement: Supplemental Information 3 [file peerj-cs-07-494-s003.zip › 0MQD6mnoy4l3zV8WPRYe.bytes.jpg]

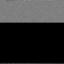

Supplement: Supplemental Information 3 [file peerj-cs-07-494-s003.zip › 0MsQ16zDg9XVerbmfCdU.bytes.jpg]

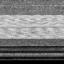

Supplement: Supplemental Information 3 [file peerj-cs-07-494-s003.zip › 0Mx7E5XgoRcJavdPAit8.bytes.jpg]

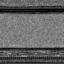

Supplement: Supplemental Information 3 [file peerj-cs-07-494-s003.zip › 0NEsQlDGnUMg3Bew7R1A.bytes.jpg]

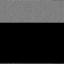

Supplement: Supplemental Information 3 [file peerj-cs-07-494-s003.zip › 0NiOTDde1ktxg954SJFE.bytes.jpg]

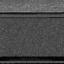

Supplement: Supplemental Information 3 [file peerj-cs-07-494-s003.zip › 0njs7MJQObCY8ABgykiP.bytes.jpg]

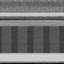

Supplement: Supplemental Information 3 [file peerj-cs-07-494-s003.zip › 0NmoAEOtIDdwiVr9PCBf.bytes.jpg]

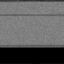

Supplement: Supplemental Information 3 [file peerj-cs-07-494-s003.zip › 0NXFnJyOEhBAISKfiU67.bytes.jpg]

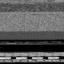

Supplement: Supplemental Information 3 [file peerj-cs-07-494-s003.zip › 0nxrvcZJUBNGM8Vg4SRf.bytes.jpg]

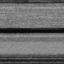

Supplement: Supplemental Information 3 [file peerj-cs-07-494-s003.zip › 0NyfGXt8nmlK72Q9Irhs.bytes.jpg]

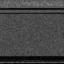

Supplement: Supplemental Information 3 [file peerj-cs-07-494-s003.zip › 0NZT2sFp4JKXqYGC8Rna.bytes.jpg]
