# Supplementary figures and images for: Malware homology determination using visualized images and feature fusion (part 3 of 4)
Source: PeerJ Comput Sci. 2021 Apr 15;7:e494. doi: 10.7717/peerj-cs.494 (PMC8056249; doi:10.7717/peerj-cs.494)

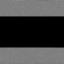

Supplement: Supplemental Information 3 [file peerj-cs-07-494-s003.zip › 04BfoQRA6XEshiNuI7pF.bytes.jpg]

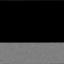

Supplement: Supplemental Information 3 [file peerj-cs-07-494-s003.zip › 04cvLCVPqBMs6yn5xGlE.bytes.jpg]

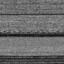

Supplement: Supplemental Information 3 [file peerj-cs-07-494-s003.zip › 04EjIdbPV5e1XroFOpiN.bytes.jpg]

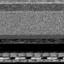

Supplement: Supplemental Information 3 [file peerj-cs-07-494-s003.zip › 04hSzLv5s2TDYPlcgpHB.bytes.jpg]

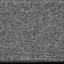

Supplement: Supplemental Information 3 [file peerj-cs-07-494-s003.zip › 04mcPSei852tgIKUwTJr.bytes.jpg]

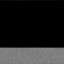

Supplement: Supplemental Information 3 [file peerj-cs-07-494-s003.zip › 04QzZ3DVdPsEp9elLR65.bytes.jpg]

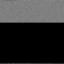

Supplement: Supplemental Information 3 [file peerj-cs-07-494-s003.zip › 04sJnMaORYc1SV5pKjrP.bytes.jpg]

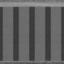

Supplement: Supplemental Information 3 [file peerj-cs-07-494-s003.zip › 05aiMRw13bYWqZ8OHvjl.bytes.jpg]

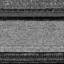

Supplement: Supplemental Information 3 [file peerj-cs-07-494-s003.zip › 05EeG39MTRrI6VY21DPd.bytes.jpg]

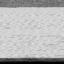

Supplement: Supplemental Information 3 [file peerj-cs-07-494-s003.zip › 05IXcWGxvnkto4sq17zZ.bytes.jpg]

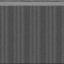

Supplement: Supplemental Information 3 [file peerj-cs-07-494-s003.zip › 05Kps4iFw8mOLJZQrb1H.bytes.jpg]

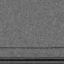

Supplement: Supplemental Information 3 [file peerj-cs-07-494-s003.zip › 05LHG8fR3iPn6agIo9z7.bytes.jpg]

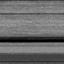

Supplement: Supplemental Information 3 [file peerj-cs-07-494-s003.zip › 05rJTUWYAKNegBk2wE8X.bytes.jpg]

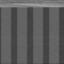

Supplement: Supplemental Information 3 [file peerj-cs-07-494-s003.zip › 065EZhxgbLRSHsB87uIF.bytes.jpg]

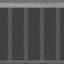

Supplement: Supplemental Information 3 [file peerj-cs-07-494-s003.zip › 06aLOj8EUXMByS423sum.bytes.jpg]

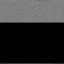

Supplement: Supplemental Information 3 [file peerj-cs-07-494-s003.zip › 06arUi9q3wHS2C8RZxeB.bytes.jpg]

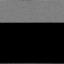

Supplement: Supplemental Information 3 [file peerj-cs-07-494-s003.zip › 06KfrF7ltESna2ZHPVp5.bytes.jpg]

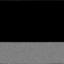

Supplement: Supplemental Information 3 [file peerj-cs-07-494-s003.zip › 06osXqPUVM1HbvBGNncT.bytes.jpg]

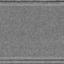

Supplement: Supplemental Information 3 [file peerj-cs-07-494-s003.zip › 06QinlpeFIWj8qHc7Vys.bytes.jpg]

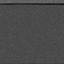

Supplement: Supplemental Information 3 [file peerj-cs-07-494-s003.zip › 07ECKjDTyQLnabNoxrIH.bytes.jpg]

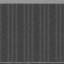

Supplement: Supplemental Information 3 [file peerj-cs-07-494-s003.zip › 07iSOIG2urUvsMl9E5Rn.bytes.jpg]

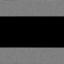

Supplement: Supplemental Information 3 [file peerj-cs-07-494-s003.zip › 07nrG1cLKUPxjOlWMFiV.bytes.jpg]

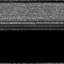

Supplement: Supplemental Information 3 [file peerj-cs-07-494-s003.zip › 08BX5Slp2I1FraZWbc6j.bytes.jpg]

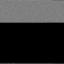

Supplement: Supplemental Information 3 [file peerj-cs-07-494-s003.zip › 09bfacpUzuBN5W3S8KTo.bytes.jpg]

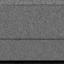

Supplement: Supplemental Information 3 [file peerj-cs-07-494-s003.zip › 09CPNMYyQjSguFrE8UOf.bytes.jpg]

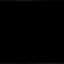

Supplement: Supplemental Information 3 [file peerj-cs-07-494-s003.zip › 09LXtWxm1EbK5uVqcQS3.bytes.jpg]

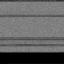

Supplement: Supplemental Information 3 [file peerj-cs-07-494-s003.zip › 09sXMJUHwQWVanrhzAoT.bytes.jpg]

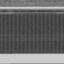

Supplement: Supplemental Information 3 [file peerj-cs-07-494-s003.zip › 0A32eTdBKayjCWhZqDOQ.bytes.jpg]

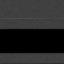

Supplement: Supplemental Information 3 [file peerj-cs-07-494-s003.zip › 0ACDbR5M3ZhBJajygTuf.bytes.jpg]

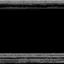

Supplement: Supplemental Information 3 [file peerj-cs-07-494-s003.zip › 0AguvpOCcaf2myVDYFGb.bytes.jpg]

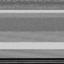

Supplement: Supplemental Information 3 [file peerj-cs-07-494-s003.zip › 0akIgwhWHYm1dzsNqBFx.bytes.jpg]

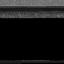

Supplement: Supplemental Information 3 [file peerj-cs-07-494-s003.zip › 0aKlH1MRxLmv34QGhEJP.bytes.jpg]

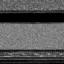

Supplement: Supplemental Information 3 [file peerj-cs-07-494-s003.zip › 0AnoOZDNbPXIr2MRBSCJ.bytes.jpg]

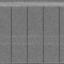

Supplement: Supplemental Information 3 [file peerj-cs-07-494-s003.zip › 0ASH2csN7k8jZyoRaqtn.bytes.jpg]

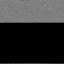

Supplement: Supplemental Information 3 [file peerj-cs-07-494-s003.zip › 0aSTGBVRXeJhx5OcpsgC.bytes.jpg]

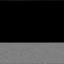

Supplement: Supplemental Information 3 [file peerj-cs-07-494-s003.zip › 0aU7XWsr8RtN94jvo3lG.bytes.jpg]

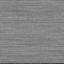

Supplement: Supplemental Information 3 [file peerj-cs-07-494-s003.zip › 0AV6MPlrTWG4fYI7NBtQ.bytes.jpg]

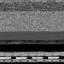

Supplement: Supplemental Information 3 [file peerj-cs-07-494-s003.zip › 0aVNj3qFgEZI6Akf4Kuv.bytes.jpg]

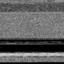

Supplement: Supplemental Information 3 [file peerj-cs-07-494-s003.zip › 0aVxkvmflEizUBG2rMT4.bytes.jpg]

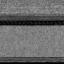

Supplement: Supplemental Information 3 [file peerj-cs-07-494-s003.zip › 0AwWs42SUQ19mI7eDcTC.bytes.jpg]

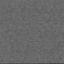

Supplement: Supplemental Information 3 [file peerj-cs-07-494-s003.zip › 0B2RwKm6dq9fjUWDNIOa.bytes.jpg]

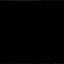

Supplement: Supplemental Information 3 [file peerj-cs-07-494-s003.zip › 0b5LqcWix3J4fGIEhXQu.bytes.jpg]

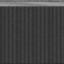

Supplement: Supplemental Information 3 [file peerj-cs-07-494-s003.zip › 0BEsCP7NAUy8XmkenHWG.bytes.jpg]

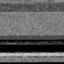

Supplement: Supplemental Information 3 [file peerj-cs-07-494-s003.zip › 0BFIPv1rO83whtpMYyAs.bytes.jpg]

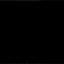

Supplement: Supplemental Information 3 [file peerj-cs-07-494-s003.zip › 0BIdbVDEgmPwjYF4xzir.bytes.jpg]

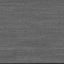

Supplement: Supplemental Information 3 [file peerj-cs-07-494-s003.zip › 0bjN3Kgw5OATSreRmEdi.bytes.jpg]

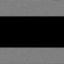

Supplement: Supplemental Information 3 [file peerj-cs-07-494-s003.zip › 0BKcmNv4iGY2hsVSaXJ6.bytes.jpg]

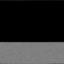

Supplement: Supplemental Information 3 [file peerj-cs-07-494-s003.zip › 0BLbmzJRkjNynCgQIdtV.bytes.jpg]

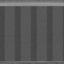

Supplement: Supplemental Information 3 [file peerj-cs-07-494-s003.zip › 0bN6ODYWw2xeCQBn3tEg.bytes.jpg]

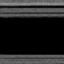

Supplement: Supplemental Information 3 [file peerj-cs-07-494-s003.zip › 0BY2iPso3bEmudlUzpfq.bytes.jpg]

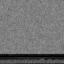

Supplement: Supplemental Information 3 [file peerj-cs-07-494-s003.zip › 0BZQIJak6Pu2tyAXfrzR.bytes.jpg]

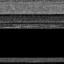

Supplement: Supplemental Information 3 [file peerj-cs-07-494-s003.zip › 0C4aVbN58O1nAigFJt9z.bytes.jpg]

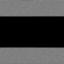

Supplement: Supplemental Information 3 [file peerj-cs-07-494-s003.zip › 0cdnSIvN489sFUwYlrMQ.bytes.jpg]

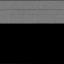

Supplement: Supplemental Information 3 [file peerj-cs-07-494-s003.zip › 0cfGJLYgE6ROaZH7KT1h.bytes.jpg]

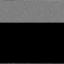

Supplement: Supplemental Information 3 [file peerj-cs-07-494-s003.zip › 0cfIE39ihRNo2rkZOw5H.bytes.jpg]

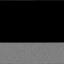

Supplement: Supplemental Information 3 [file peerj-cs-07-494-s003.zip › 0cGWK6VvCkm7O2AxDjtw.bytes.jpg]

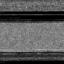

Supplement: Supplemental Information 3 [file peerj-cs-07-494-s003.zip › 0cH8YeO15ZywEhPrJvmj.bytes.jpg]

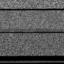

Supplement: Supplemental Information 3 [file peerj-cs-07-494-s003.zip › 0co46B8IkPt2UN3HSaw7.bytes.jpg]

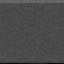

Supplement: Supplemental Information 3 [file peerj-cs-07-494-s003.zip › 0CPaAXtyswrBq83D6VEg.bytes.jpg]

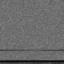

Supplement: Supplemental Information 3 [file peerj-cs-07-494-s003.zip › 0Cq4wfhLrKBJiut1lYAZ.bytes.jpg]

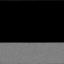

Supplement: Supplemental Information 3 [file peerj-cs-07-494-s003.zip › 0csgzpwdL3FbZEJu6DjO.bytes.jpg]

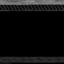

Supplement: Supplemental Information 3 [file peerj-cs-07-494-s003.zip › 0cTu2bkefOAJqIhYUWFK.bytes.jpg]

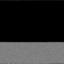

Supplement: Supplemental Information 3 [file peerj-cs-07-494-s003.zip › 0CzL6rfwaTqGOu9eghBt.bytes.jpg]

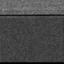

Supplement: Supplemental Information 3 [file peerj-cs-07-494-s003.zip › 0czUXKSCiGY2j5mxLdWa.bytes.jpg]

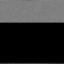

Supplement: Supplemental Information 3 [file peerj-cs-07-494-s003.zip › 0D9IedmC1viTPugLRWX6.bytes.jpg]

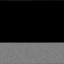

Supplement: Supplemental Information 3 [file peerj-cs-07-494-s003.zip › 0daTri9PSkeEsVHu5Dhw.bytes.jpg]

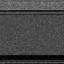

Supplement: Supplemental Information 3 [file peerj-cs-07-494-s003.zip › 0dauMIK4ATfybzqUgNLc.bytes.jpg]

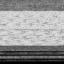

Supplement: Supplemental Information 3 [file peerj-cs-07-494-s003.zip › 0DbLeKSoxu47wjqVHsi9.bytes.jpg]

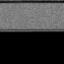

Supplement: Supplemental Information 3 [file peerj-cs-07-494-s003.zip › 0df4cbsTBCn1VGW8lQRv.bytes.jpg]

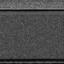

Supplement: Supplemental Information 3 [file peerj-cs-07-494-s003.zip › 0dhL8Jvcswa7U1qHiDS5.bytes.jpg]

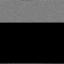

Supplement: Supplemental Information 3 [file peerj-cs-07-494-s003.zip › 0Dk7Wd8MERu3b5rmQzCK.bytes.jpg]

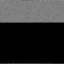

Supplement: Supplemental Information 3 [file peerj-cs-07-494-s003.zip › 0dkuzUXLTEFwW71vP5bS.bytes.jpg]

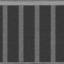

Supplement: Supplemental Information 3 [file peerj-cs-07-494-s003.zip › 0DM3hS6Gg2QVKb1fZydv.bytes.jpg]

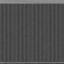

Supplement: Supplemental Information 3 [file peerj-cs-07-494-s003.zip › 0dnTixlMYzDUpsvEVrGc.bytes.jpg]

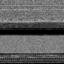

Supplement: Supplemental Information 3 [file peerj-cs-07-494-s003.zip › 0DNVFKwYlcjO7bTfJ5p1.bytes.jpg]

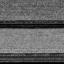

Supplement: Supplemental Information 3 [file peerj-cs-07-494-s003.zip › 0DqUX5rkg3IbMY6BLGCE.bytes.jpg]

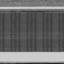

Supplement: Supplemental Information 3 [file peerj-cs-07-494-s003.zip › 0DTp59Av1RLifoKlUdm7.bytes.jpg]

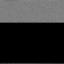

Supplement: Supplemental Information 3 [file peerj-cs-07-494-s003.zip › 0DTs2PhZfCwEv7q8349K.bytes.jpg]

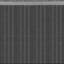

Supplement: Supplemental Information 3 [file peerj-cs-07-494-s003.zip › 0EAdHtLDypMcwjTFJziC.bytes.jpg]

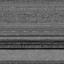

Supplement: Supplemental Information 3 [file peerj-cs-07-494-s003.zip › 0eaNKwluUmkYdIvZ923c.bytes.jpg]

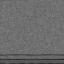

Supplement: Supplemental Information 3 [file peerj-cs-07-494-s003.zip › 0EL7OGZKozbiNCVP61gk.bytes.jpg]

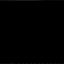

Supplement: Supplemental Information 3 [file peerj-cs-07-494-s003.zip › 0eN9lyQfwmTVk7C2ZoYp.bytes.jpg]

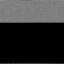

Supplement: Supplemental Information 3 [file peerj-cs-07-494-s003.zip › 0Eo9qT6idXHDMebwmvPA.bytes.jpg]

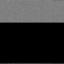

Supplement: Supplemental Information 3 [file peerj-cs-07-494-s003.zip › 0evDQX7AVfC1ZTJEKltg.bytes.jpg]

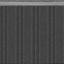

Supplement: Supplemental Information 3 [file peerj-cs-07-494-s003.zip › 0F4qIHaR7xOrm19Set3o.bytes.jpg]

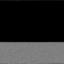

Supplement: Supplemental Information 3 [file peerj-cs-07-494-s003.zip › 0FdOaDWrfBU6TqwCRYxA.bytes.jpg]

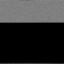

Supplement: Supplemental Information 3 [file peerj-cs-07-494-s003.zip › 0fGuCWgTraQ6nEmLPN8q.bytes.jpg]

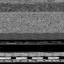

Supplement: Supplemental Information 3 [file peerj-cs-07-494-s003.zip › 0fhnXI9ESr4jgWmkiaTe.bytes.jpg]

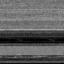

Supplement: Supplemental Information 3 [file peerj-cs-07-494-s003.zip › 0fHVZKeTE6iRb1PIQ4au.bytes.jpg]

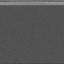

Supplement: Supplemental Information 3 [file peerj-cs-07-494-s003.zip › 0FKerJl18xOc3jdoyg4A.bytes.jpg]

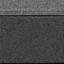

Supplement: Supplemental Information 3 [file peerj-cs-07-494-s003.zip › 0FOXjzmnD9CUMVcSlEqh.bytes.jpg]

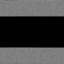

Supplement: Supplemental Information 3 [file peerj-cs-07-494-s003.zip › 0Fu9oETtMW4zlg1ZrUy6.bytes.jpg]

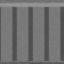

Supplement: Supplemental Information 3 [file peerj-cs-07-494-s003.zip › 0fvnGU7dkbr8iEhZuMcP.bytes.jpg]

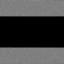

Supplement: Supplemental Information 3 [file peerj-cs-07-494-s003.zip › 0fxgjYEClPL1BDbcshzJ.bytes.jpg]

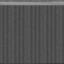

Supplement: Supplemental Information 3 [file peerj-cs-07-494-s003.zip › 0G2RV1chBlIbkt6JqA5Q.bytes.jpg]

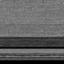

Supplement: Supplemental Information 3 [file peerj-cs-07-494-s003.zip › 0G4hwobLuAzvl1PWYfmd.bytes.jpg]

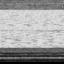

Supplement: Supplemental Information 3 [file peerj-cs-07-494-s003.zip › 0GbMkYlNyt72OzBjIcVh.bytes.jpg]

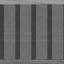

Supplement: Supplemental Information 3 [file peerj-cs-07-494-s003.zip › 0gCmlyxw2UJvX7SNOGqu.bytes.jpg]

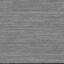

Supplement: Supplemental Information 3 [file peerj-cs-07-494-s003.zip › 0gcZkSFr7VnEmLPbTxUe.bytes.jpg]

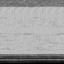

Supplement: Supplemental Information 3 [file peerj-cs-07-494-s003.zip › 0gDsIvrylX5fPbG7cSBn.bytes.jpg]
